# The homeostatic dynamics of feeding behaviour identify novel mechanisms of anorectic agents

**DOI:** 10.1371/journal.pbio.3000482

**Published:** 2019-12-05

**Authors:** Thomas M. McGrath, Eleanor Spreckley, Aina Fernandez Rodriguez, Carlo Viscomi, Amin Alamshah, Elina Akalestou, Kevin G. Murphy, Nick S. Jones

**Affiliations:** 1 Department of Mathematics, Imperial College London, London, United Kingdom; 2 EPSRC Centre for the Mathematics of Precision Healthcare, Imperial College London, London, United Kingdom; 3 Section of Endocrinology and Investigative Medicine, Imperial College London, London, United Kingdom; 4 MRC Mitochondrial Biology Unit, University of Cambridge, Cambridge, United Kingdom; University of Southern California, UNITED STATES

## Abstract

Better understanding of feeding behaviour will be vital in reducing obesity and metabolic syndrome, but we lack a standard model that captures the complexity of feeding behaviour. We construct an accurate stochastic model of rodent feeding at the bout level in order to perform quantitative behavioural analysis. Analysing the different effects on feeding behaviour of peptide YY3-36 (PYY3-36), lithium chloride, glucagon-like peptide 1 (GLP-1), and leptin shows the precise behavioural changes caused by each anorectic agent. Our analysis demonstrates that the changes in feeding behaviour evoked by the anorectic agents investigated do not mimic the behaviour of well-fed animals and that the intermeal interval is influenced by fullness. We show how robust homeostatic control of feeding thwarts attempts to reduce food intake and how this might be overcome. In silico experiments suggest that introducing a minimum intermeal interval or modulating upper gut emptying can be as effective as anorectic drug administration.

## Abstract 2

A quantitative study of rodent feeding behaviour reveals that anorectic agents may have substantially different behavioural effects from one another and from the satiated state they are intended to evoke. In silico studies suggest alternative strategies for reducing food intake.

## Introduction

The current obesity epidemic has driven significant interest in developing novel therapies to reduce food intake. However, the homeostatic nature of energy balance makes feeding a difficult process to control; manipulating one aspect of feeding behaviour can result in compensatory changes in other aspects, such that it is possibly to radically restructure feeding without altering total food intake [1–3]. A more detailed understanding of how feeding behaviour is structured might allow understanding of which aspects may most promisingly be targeted to prevent or treat obesity. One natural avenue of investigation is to look in greater detail at feeding behaviour: how feeding plays out through time at the individual level, paying attention to behaviour at finer-grained temporal resolution. A natural resolution is the level of individual feeding bouts and their organisation into meals [4,5], which is conventionally referred to as microstructure analysis [6]. In this paper, we have analysed the microstructure of feeding behaviour by developing a mathematical model of food intake, which we then use to carry out a detailed examination of feeding behaviour using bout-level data and the way in which the gut governs the dynamics of feeding. This model provides novel, to our knowledge, insight into the mechanisms by which anorectic agents act to alter feeding behaviour and how food intake changes with nutritional status and photoperiod.

Modelling homeostatic behaviours such as feeding poses a particular challenge. Although recent advances in automated monitoring technology can create large data sets through quantitative, high-throughput capture and annotation of behaviour [7–12], homeostatic behaviours are driven by the need to regulate a (typically unobserved and continuously varying) variable such as body temperature or energy balance. Thus, we expect the rates of transitions between behavioural states to be strongly modulated by the variable under regulation, which we refer to as the physiological state. Conventional behavioural analysis makes heavy use of the ethogram, which charts the rates of transitions between different behaviours. However, when these rates vary when the physiological state changes, this is no longer possible, and new techniques are needed. In this paper, we suggest such a technique: a class of models known as piecewise deterministic Markov processes [13]. These generalise Markov chains to include exactly the kind of deterministically varying transition rates required by models of homeostatic behaviour and have found a wide variety of uses in biology [14–17] and physics [18,19], although they have not previously been used to study food intake or energy balance.

We use piecewise deterministic Markov processes to create a flexible and intuitive stochastic model of feeding behaviour governed by an integrated measure of recent feeding. This measure, which we will refer to as ‘fullness’, is based on a simple model of gut filling and emptying and is intended to correspond to the fullness of the stomach and small intestine. Thus, when we refer to ‘gut motility’, we mean specifically the rate at which the stomach and small intestine clear ingested food. Our model, which includes both behavioural and physiological elements, allows us to both infer characteristics of feeding behaviour both for individuals and groups of animals and to generate representative behaviour sequences in silico for a previously characterised group or individual. Although the model we introduce in this work only considers the effect of fullness on feeding behaviour and how this is modulated by anorectic agents, fasting and the day/night cycle, it can easily be extended to incorporate other factors that are known to be important such as food palatability, energy expenditure, or social factors. This could be achieved either by introducing new variables in the physiological state (e.g., in the case of energy expenditure) or by extending the current analysis to include groups in new conditions (e.g., with access to more palatable chow). Social factors influencing behaviour have previously been modelled within the mathematical framework we use [17], demonstrating the flexibility of this class of models.

The only previous generative model of feeding known to the authors is [20], which does not allow for inference from data for either individuals or groups. Previous attempts to characterise the effects of food intake on feeding behaviour have centred on the satiety ratio, which measures how the size of a meal affects the intermeal interval that follows it (see S1 Text, Section 7). We show that the satiety ratio cannot successfully model feeding behaviour because it ignores the effects of earlier meals. Furthermore, it does not define a generative model, preventing in silico experimentation. In this paper, we undertake a quantitative study of feeding microstructure in rats in a wide variety of conditions with the aim of better understanding how they affect behaviour. We generated data from rats in both photoperiods, both fasted and fed ad libitum, and given a range of anorectic drugs. In order to investigate the wider validity of the model, we also generated data from mice fed ad libitum in both the light and dark periods. These data, when combined with our model, allowed us to identify novel, to our knowledge, mechanisms of behavioural action for anorectic drugs, replicate recent in vivo results in silico, and investigate how behavioural interventions can combine with anorectic drug administration to robustly reduce food intake.

## Results

### Stochastic modelling for high-resolution feeding data

**Fig 1:**
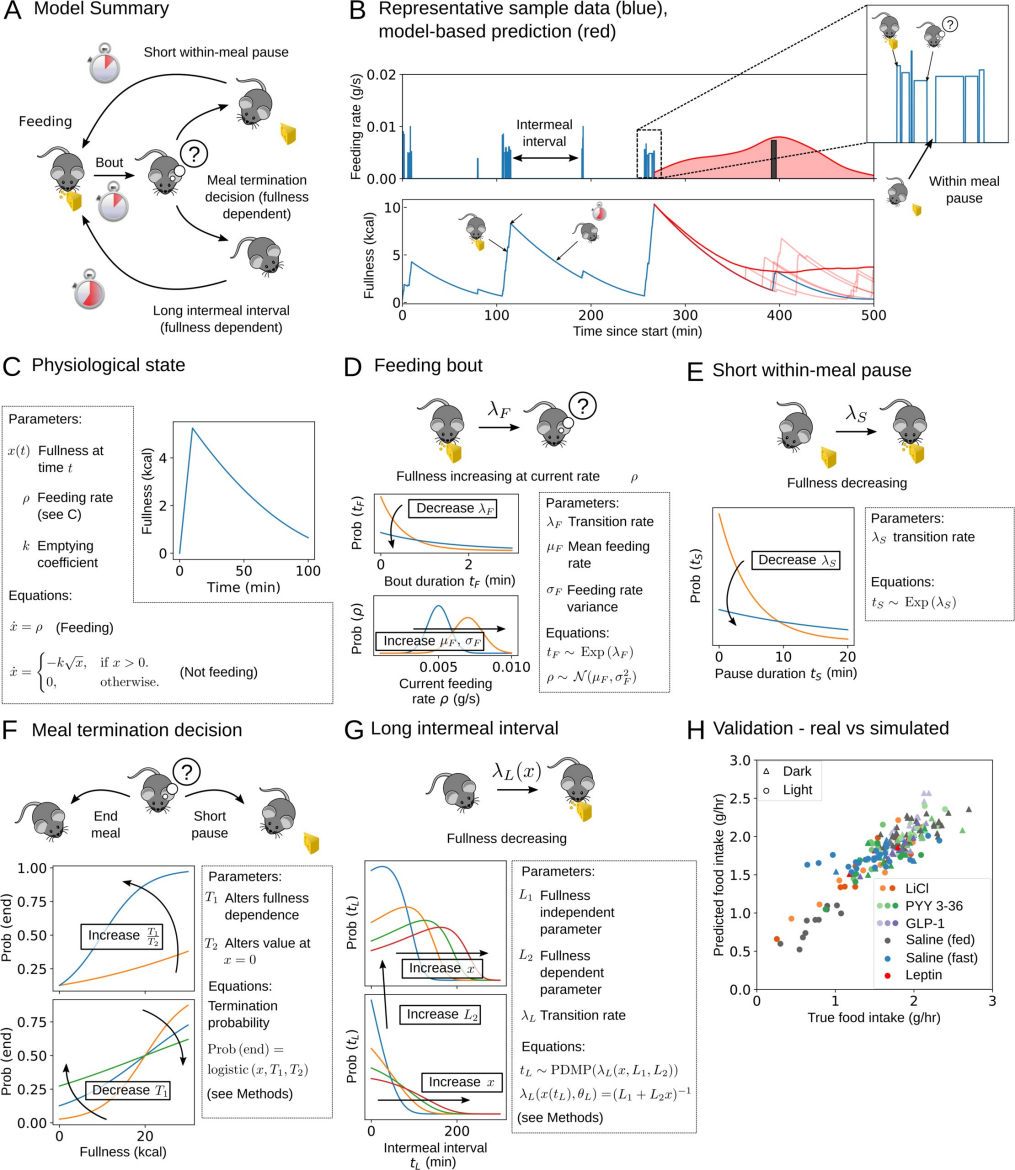
A simple stochastic model of rat feeding that uses a continuously time-varying fullness can accurately recapitulate food intake. (A) Schematic illustration of the model, showing feeding, a short within-meal pause, a long intermeal interval, and the meal termination decision, which relies on fullness *x*. (B) Incorporating fullness into a stochastic model of feeding allows prediction of the intermeal interval. The model predicts a distribution over possible intermeal interval lengths (red curve in top panel, actual next mealtime shown as a dark bar). Representative sample of data showing bout-level feeding data (top panel, blue bars) and model-derived fullness (bottom panel, blue curve). Simulated trajectories of fullness are shown in the bottom panel (light red curves) alongside mean fullness (dark red curve). Inset: bout-level feeding data for the meal shown inside the dashed box. (C) Filling occurs linearly at a rate *ρ* that varies between bouts (see D), whereas emptying is nonlinear but has identical dynamics whenever the animal is not feeding. (D) Feeding bout duration is exponentially distributed, and rate *ρ* is normally distributed with mean *μ*_*F*_ and standard deviation *σ*_*F*_. (E) Within-meal pauses are typically short compared to intermeal intervals and are distributed exponentially. Figure shows exponential distribution for representative parameter values. (F) Meal termination decisions are sigmoid in fullness *x* and are controlled by parameters *T*_1_ and *T*_2_. Keeping the product of these parameters fixed changes the effect of fullness on termination probability. (G) Intermeal intervals are typically long and depend on fullness *x*, which varies over time. Parameter *L*_1_ controls intermeal interval duration independent of fullness, and *L*_2_ measures the effect of fullness. (H) Our model accurately captures food intake: (posterior) predictive values of normalised food intake are strongly correlated with true values. Marker style indicates anorectic agent (if any) and photoperiod (see legend). Darker colours indicate higher doses; see Table 2 for concentrations. Underlying data are available in the following repository: doi:10.17632/vpm89vrz7g.1. Inference procedure described in Materials and Methods. GLP-1, glucagon-like peptide 1; PYY3-36, peptide YY3-36.

We aim to model the ways in which signals from the gut govern feeding behaviour in terms of its behavioural components, as well as how anorectic agents affect feeding behaviour and the degree of interindividual variation in behaviour. The components of feeding we consider are ingestive behaviour (the duration of feeding bouts and the rate at which food is consumed within them), the intermeal interval (satiety), and meal termination decisions (satiation). In order to do this, we construct a stochastic model of feeding behaviour, which we parametrise using a wide range of experimental data. Our model, which is summarised in Fig 1A, has two elements: a behavioural element, which determines whether the animal is feeding, pausing between bouts, or in an intermeal interval; and a physiological element, which models the filling and emptying of the gut in response to the animal's behaviour.

The physiological element of our model uses a simple one-compartment model of the gut, in which the gut fills during eating and empties between feeding bouts (Fig 1C). The variable *x* we will refer to as ‘fullness’ and should be understood to denote the contents of the stomach and the small intestine. The rate of filling can be determined directly from bout data, but in order to determine fullness at some later time after the feeding bout has ended, a model for its dynamics is needed. We model fullness as decreasing at a rate proportional to the square root of its contents (as used in [20]); however, we have also conducted a model robustness check that demonstrated that our findings are qualitatively robust to model perturbations (see S1 Text, Section 10).

The effect of fullness on feeding behaviour can be mediated by a variety of factors that depend on the part of the gut under consideration, including the release of gut peptides, mechanoreception, and signalling via the vagus nerve [21,22]. Our model should be understood as an integrative model that combines the effects of all of these factors in the behavioural element. We outline possible expansions of the model to include more detailed physiological variables in the Discussion. As we will show, even a simple model with a single fullness variable has substantial predictive power and is able to both capture the dynamics of feeding behaviour and provide insight into the effects of anorectic agents. One of the contributions of our work is to provide a ‘minimal model’ of feeding behaviour, which is sufficient to provide new insights but can be extended and developed in greater detail.

The behavioural element of our model uses the conventional partitioning of feeding into bouts and meals: within a meal, bouts are punctuated by short pauses, with longer intermeal intervals occupying the time between meals. A number of behavioural correlates, including the behavioural satiety sequence, indicate that this is a meaningful distinction and that intermeal intervals are not simply unusually long interbout pauses [4]. We used bout-level feeding data obtained from the Columbus Instruments Comprehensive Lab Animal-Monitoring System (CLAMS), although other systems such as BioDAQ can provide suitable data. These data were then used as input to a simple model of gut filling and emptying [20] to provide a continuous measurement of fullness through time, as shown in Fig 1B and 1C. To summarise, our model contains two parts: a physiological state consisting of a model of gut filling and emptying (Fig 1C) and a model of transitions between behavioural states (Fig 1D–1G). In a given behavioural state, the physiological state evolves deterministically; e.g., during a feeding bout, the animal's gut fills at a constant rate. The behavioural state, however, switches stochastically, and its probability of transitioning from one state to another at any given moment depends on the physiological state at that moment. Our model is therefore capable of capturing homeostatic behaviour.

The dynamics of our model can be summarised as follows: a feeding bout occurs with stochastic duration and feeding rate. This increases fullness. Following the termination of this feeding bout, a meal termination decision is made that is also stochastic but depends on fullness. If the meal is not terminated, a short within-meal pause occurs whose duration is stochastic but independent of fullness. If the meal is terminated, however, then a long intermeal interval occurs, whose duration depends on fullness. The distribution of each stochastic event is controlled by parameters that vary between rats but are fixed for each individual in a group (nutritional state, drug administration, and photoperiod), summarised in Table 1. The degree of variation between rats in the same group is controlled by group-level parameters *μ* and Σ, summarised in the same table. These parameters define the ‘typical’ rat in the group, as well as how much rats within the group vary.

**Table 1:** Summary and description of model parameters. See Fig 1 for a graphical summary and equations.

Parameter	Description	Comments
**Feeding bout**		
*λ*_*F*_	Feeding bout termination rate	Probability of feeding bout ending per unit time—sets mean bout duration
*ρ*_*F*_	Feeding rate	Rate of feeding in a bout—randomly sampled for each bout
*μ*_*F*_	Mean bout feeding rate	Bout feeding rates are normally distributed for each animal—this sets the mean
*σ*_*F*_	Feeding rate variance	Sets the variance of the normal distribution of feeding rates
**Short within-meal pause**		
*λ*_*S*_	Short pause termination rate	Probability of a short within-meal pause ending per unit time—sets mean short pause duration
**Meal termination**		
*T*_1_	Meal termination parameter 1	Makes meal termination probability curve sharper (see Fig 1F)
*T*_2_	Meal termination parameter 2	Makes meal termination more fullness-sensitive (see Fig 1F)
**Intermeal interval**		
*L*_1_	Intermeal interval parameter 1	Sets fullness-independent component of intermeal interval termination rate (see Fig 1G)
*L*_2_	Intermeal interval parameter 2	Sets fullness-dependent component of intermeal interval termination rate (see Fig 1G)
**Group characteristics**		
Θ	Group mean	Defines the ‘typical individual’ for a group—the mean of the group distribution
Σ	Group covariance matrix	Sets the interindividual variation of parameters and how correlated different parameters are
**Other**		
*k*	Gut motility parameter	Sets rate of decrease of fullness via $\dot{x}=-k\sqrt{x}$ (where *x* is fullness)

### Our stochastic model can accurately capture rat feeding behaviour in a wide variety of conditions

We carried out a series of experimental studies in order to obtain behavioural data from rats given low, medium, and high doses of peptide YY3-36 (PYY3-36) in the light and dark period, as well as rats given low and high doses of lithium chloride (LiCl) in the light period and rats given low, medium, and high doses of glucagon-like peptide 1 (GLP-1) and leptin in the dark period (details and doses in Table 2 and Materials and Methods). From these experiments, we also obtained control data from rats given saline under each of these conditions. All experiments in the light period were carried out following an overnight fast, whereas dark period experiments involved ad libitum feeding. In addition, we carried out a longer duration feeding study over three days with untreated rats recovering from a fast, providing further data on ‘natural’ behavioural patterns. We further obtained bout-level feeding data from mice in order to determine whether our model had a wider range of applicability. This exploratory analysis and details of data collection and processing are detailed in S1 Text, Section 4. We found that with minimal reparametrisation in order to account for the different total food intake in mice, we were able to model mouse feeding behaviour and recapitulate a novel tradeoff in ingestive behaviour that we discovered in rats (see below).

**Table 2:** Details of drug doses and administration protocols. Light on/off times were 06:00 and 18:00, respectively. Anorectic agents were GLP-1, PYY3-36, lithium chloride (LiCl), and leptin.

Drug	Dose	Start Time	Duration	Prior Feeding	# Animals	Protocol
GLP-1	30 nmol/kg	19:00	8 hours	ad libitum	8	1
GLP-1	100 nmol/kg	19:00	8 hours	ad libitum	8	1
GLP-1	300 nmol/kg	19:00	8 hours	ad libitum	9	1
PYY3-36	1.5 nmol/kg	19:00	8 hours	ad libitum	12	1
PYY3-36	100 nmol/kg	19:00	8 hours	ad libitum	11	1
PYY3-36	300 nmol/kg	19:00	8 hours	ad libitum	14	1
Leptin	2 mg/kg	19:00	8 hours	ad libitum	6	1
Saline	n/a	19:00	8 hours	ad libitum	24	1
LiCl	32 mg/kg	09:00	8 hours	fasted overnight	10	2
LiCl	64 mg/kg	09:00	8 hours	fasted overnight	9	2
PYY3-36	1.5 nmol/kg	09:00	8 hours	fasted overnight	8	2
PYY3-36	100 nmol/kg	09:00	8 hours	fasted overnight	6	2
PYY3-36	300 nmol/kg	09:00	8 hours	fasted overnight	9	2
Saline	n/a	09:00	8 hours	fasted overnight	13	2
Saline	n/a	06:00	10 hours	fasted overnight	11	3
Saline	n/a	18:00	10 hours	fasted overnight	11	3
Saline	n/a	06:00	10 hours (× 2)	ad libitum	11	3
Saline	n/a	18:00	10 hours (× 2)	ad libitum	11	3

**Abbreviations:** GLP-1, glucagon-like peptide 1; n/a, not applicable; PYY3-36, peptide YY3-36.

After fitting the model to the data (see Materials and Methods), we validated the model by simulating behaviour to provide predictions about the next mealtime, total food consumption, or future fullness, as shown in Fig 1B. A subset of the saline and PYY3-36 data was used in model design; however, the model was further validated by comparing fits to unseen saline data with fits to saline data from identical conditions (see S1 Text, Section 3). The long-term organisation of feeding behaviour into meals and bouts occurs on timescales comparable to typical experimental observations: we may only see 5–10 meals for a single rat's time series. This complicates inference if we also want to consider the possibility of interindividual variation. We used Bayesian hierarchical modelling [23, 24] to allow pooling of data across individuals in a statistically rigorous way and to infer the degree of interindividual variation (see Materials and Methods). This inference procedure produced a distribution over parameters for individual rats and for groups of rats; these parameters are summarised in Table 1. Unless otherwise stated, this distribution was used to produce the subsequent results. This parameter distribution can be resampled using the data and inference code from the link below.

In order to verify the model's predictive power, we generated predictions for food intake for each individual by repeatedly Monte Carlo sampling using parameters drawn from individual-level posteriors (see S1 Text, Section 3). Our predictions agreed with observed food intake across a wide range of food intakes from different photoperiods, anorectic drugs, and nutritional states (as shown in Fig 1H) in spite of the level of stochasticity inherent in feeding, confirming that our model captured food intake accurately. Code for inference, figure generation, and a set of simple tools for behavioural modelling are available at https://github.com/tomMcGrath/feeding-behaviour, and full details of drug administration doses and protocols are given in Table 2.

### Both within-bout and intermeal behaviour determine food intake

**Fig 2:**
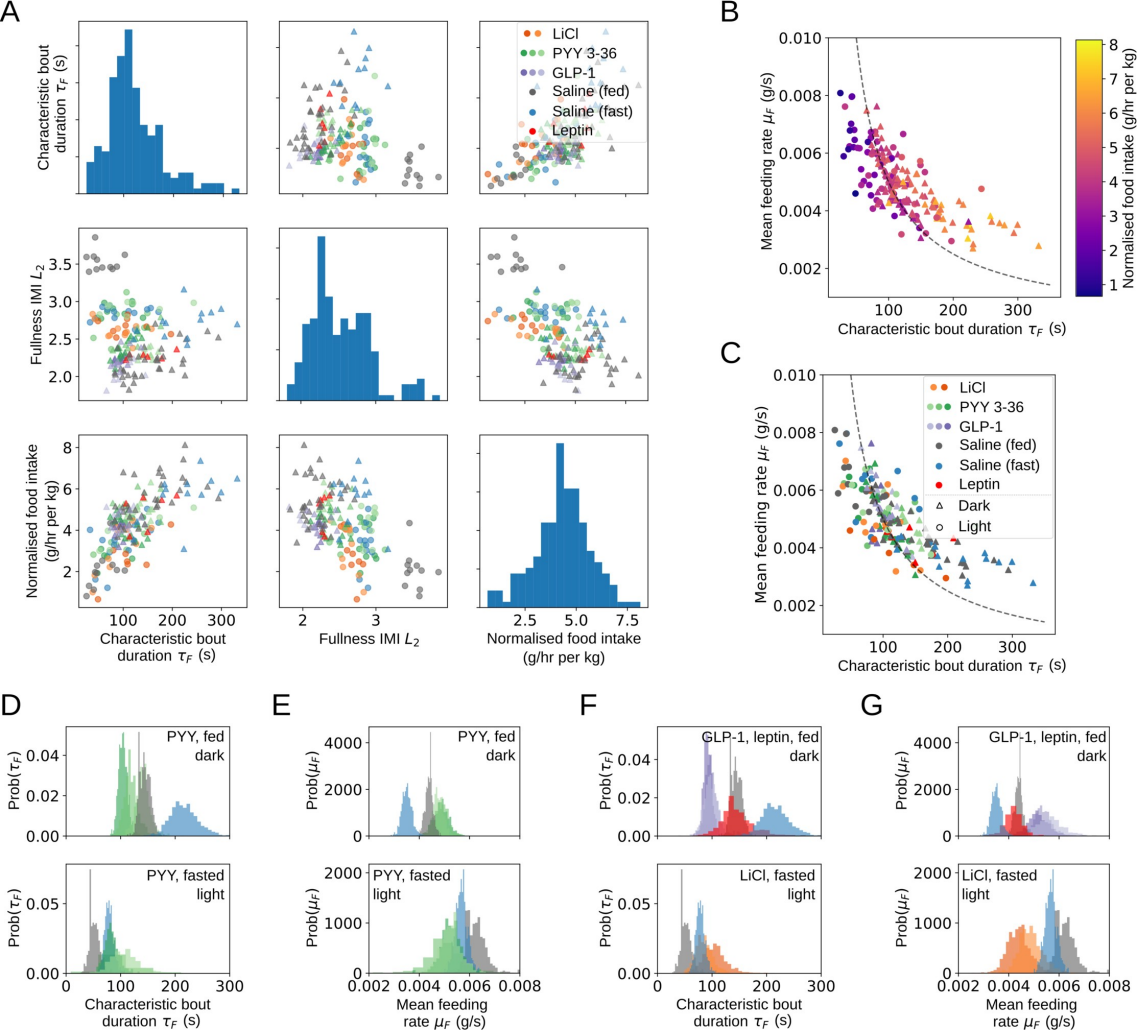
A two-parameter behavioural fingerprint, combining characteristic bout duration and intermeal interval parameters, accounts for a large proportion of variation both between and within groups. Different anorectic agents drive different patterns of feeding behaviour. (A) Two parameters of the model are strongly informative regarding food intake but are only weakly correlated with one another: characteristic bout duration *τ*_*F*_ and fullness-dependent intermeal interval parameter *L*_2_. Scatter matrix of *τ*_*F*_, *L*_2_, and normalised food intake (diagonal entries show univariate marginals) indicates substantial intergroup variation in *τ*_*F*_ and *L*_2_ correlated with changes in normalised food intake. (B) Reduced characteristic bout durations are not fully compensated for by increased feeding rate: scatter plot of individual posterior mean values coloured by normalised food intake. Dashed line indicates constant food intake contour. (C) Characteristic bout duration and feeding rate vary both within and between groups. Animals in the dark period tend to have longer, slower bouts. (D, E) PYY3-36 causes group-level variation in bout time and feeding rate. Colours as in Fig 2C. Animals recovering from an overnight fast show less variation than rats fed ad libitum. (F, G) GLP-1 produces a more pronounced effect on both bout duration and rate than PYY3-36 in rats fed ad libitum. Lithium chloride produces a stronger reduction in feeding rate than PYY3-36 in rats recovering from an overnight fast but has limited effect on bout duration. Underlying data are available in the following repository: doi:10.17632/vpm89vrz7g.1. Inference procedure described in Materials and Methods. GLP-1, glucagon-like peptide 1; IMI, intermeal interval; PYY3-36, peptide YY3-36.

When each of the model parameters were compared with mean hourly food intake, two stood out as clear determinants of food intake: mean bout duration *τ*_*F*_ = 1/*λ*_*F*_ and the parameter governing the dependence of the intermeal interval on fullness *L*_2_ (Fig 2A). Bout duration and fullness-dependent intermeal interval are strongly related to food intake: decreasing the former leads to shorter meals, and increasing the latter means that fullness strongly suppresses meal initiation, leading to long intermeal intervals in most circumstances. These two important parameters are found to be only weakly correlated with one another but are both strongly correlated with food intake (Fig 2A), making them an informative way to determine what behavioural changes may have led to an observed change in food intake. The fullness-dependent intermeal interval parameter *L*_2_ shows correlation with food intake across but not within groups, suggesting that although different conditions and anorectic agents can cause substantial changes in intermeal interval, rats exhibit limited interindividual variability along this axis, unlike the considerable interindividual variation in ingestive behaviour (Fig 2A). The within-group variation in food intake at similar values of *L*_2_ is likely to be due to the natural variability of food intake even when parameters are held constant (see Fig 4C), whereas the between-group variation is due to differences in other behavioural parameters.

### Ingestive behaviour has a single axis of variation that is modulated by anorectic drugs, photoperiod, and nutritional status

In order to consume more food in a given bout, an animal can either eat for longer or it can eat faster. In principle, bout duration and feeding rate could trade off exactly to keep the amount consumed in a bout the same for any bout duration or could be completely uncorrelated. In practice we found that feeding rate imperfectly compensates for reduced bout duration: shorter bout durations were associated with decreased food intake (Fig 2B and 2C) both within and across groups (see S1 Text, Section 6). Analysis of feeding data from mice fed ad libitum in the light and dark period showed an identical pattern (see S2 Fig), indicating that this tradeoff may apply more generally. Although feeding rate increased at shorter bout durations, it did not do so sufficiently to compensate for the decreased time available to feed, leading to reduced food intake. Longer, slower bouts typically occurred during the dark period, but bout duration and feeding rate showed considerable variation between individuals, drug treatments and between fasted and rats fed ad libitum (Fig 2B and 2C). In order to understand the typical effects of anorectic agents on ingestive behaviour, we looked at the group-level posterior probabilities for bout time and feeding rate (Fig 2D–2G). These show a probability distribution over the possible values of characteristic feeding rates and bout durations for each condition. We found that PYY3-36, LiCl, and GLP-1 had a robust effect on feeding rate and bout duration at all doses and in both the light and dark period. Rats fed ad libitum have very distinct ingestive behaviours in the dark and light periods, corresponding to long, slow feeding bouts and short, fast bouts, respectively. In a pattern that was found to recur again in this analysis, the effects of anorectic agents were to reduce the difference between these behaviour modes: the day/night difference in parameters of rats given anorectic agents is less than that in rats fed ad libitum. The effect of nutritional status was the same in both photoperiods: bout duration was increased and feeding rate decreased in rats fed ad libitum compared to fasted rats during both the dark and light periods. Only LiCl showed a dose-dependent effect on bout duration and feeding rate across the concentrations administered. Of the anorectic drugs administered, GLP-1 had the most pronounced effect on feeding rate, whereas leptin had no effect on either bout duration or feeding rate. This demonstrates that although some of the anorectic agents we investigated do affect ingestive behaviour, their effect is not to mimic the feeding behaviour of animals fed ad libitum, but rather, to induce a new behavioural state.

### Fasting and photoperiod strongly affect intermeal interval by altering the effect of fullness, unlike most anorectic agents

**Fig 3:**
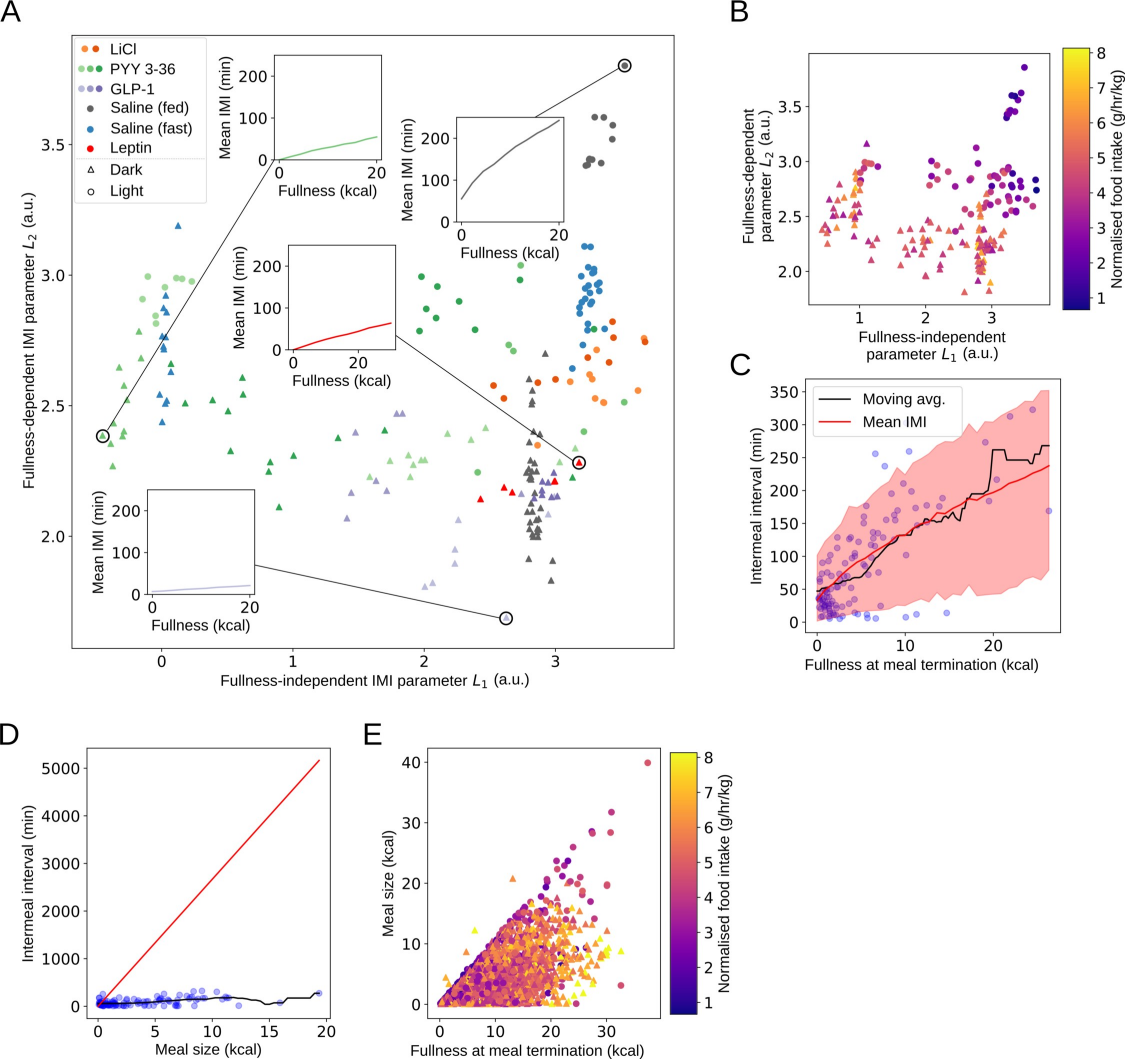
Fullness is predictive of intermeal interval; however, the relationship between fullness and intermeal interval varies with photoperiod, fasting status, and anorectic drug administration. The satiety ratio fails to capture the effect of feeding on intermeal interval because it neglects feeding prior to the most recent meal. (A) Intermeal interval parameters are affected strongly by refeeding status, photoperiod, and anorectic drug administration. Intermeal intervals of rats in the light period are strongly affected by fullness, whereas rats in the dark period have briefer intermeal intervals that are less strongly affected by fullness. Rats given lithium chloride behave similarly in the light and dark period. Rats given PYY3-36 behave more like fasted rats, whereas rats given GLP-1 and leptin have intermeal interval parameters similar to those of rats fed ad libitum. Inset figures show how parameter variation affects intermeal interval. (B) Intermeal interval parameters are strongly associated with variation in food intake: animals with lower parameters eat more. Points coloured by normalised food intake. (C) Mean (solid red line) and 95% posterior predictive interval for rats fed ad libitum given saline in the light period. Intermeal interval tracks gut emptying. Although fullness is indicative of intermeal interval, the predictive window is relatively wide. Blue circles are group data, and black line is moving window average. (D) Satiety-ratio–based predictions of intermeal interval from meal size are inaccurate. Solid red line shows satiety ratio prediction, blue circles are group data, and black line is moving window average. (E) Fullness at meal termination is poorly correlated with meal size, leading to poor predictive ability. Underlying data are available in the following repository: doi:10.17632/vpm89vrz7g.1. Inference procedure described in Materials and Methods. a.u., arbitrary unit; GLP-1, glucagon-like peptide 1; IMI, intermeal interval; PYY3-36, peptide YY3-36.

**Fig 4:**
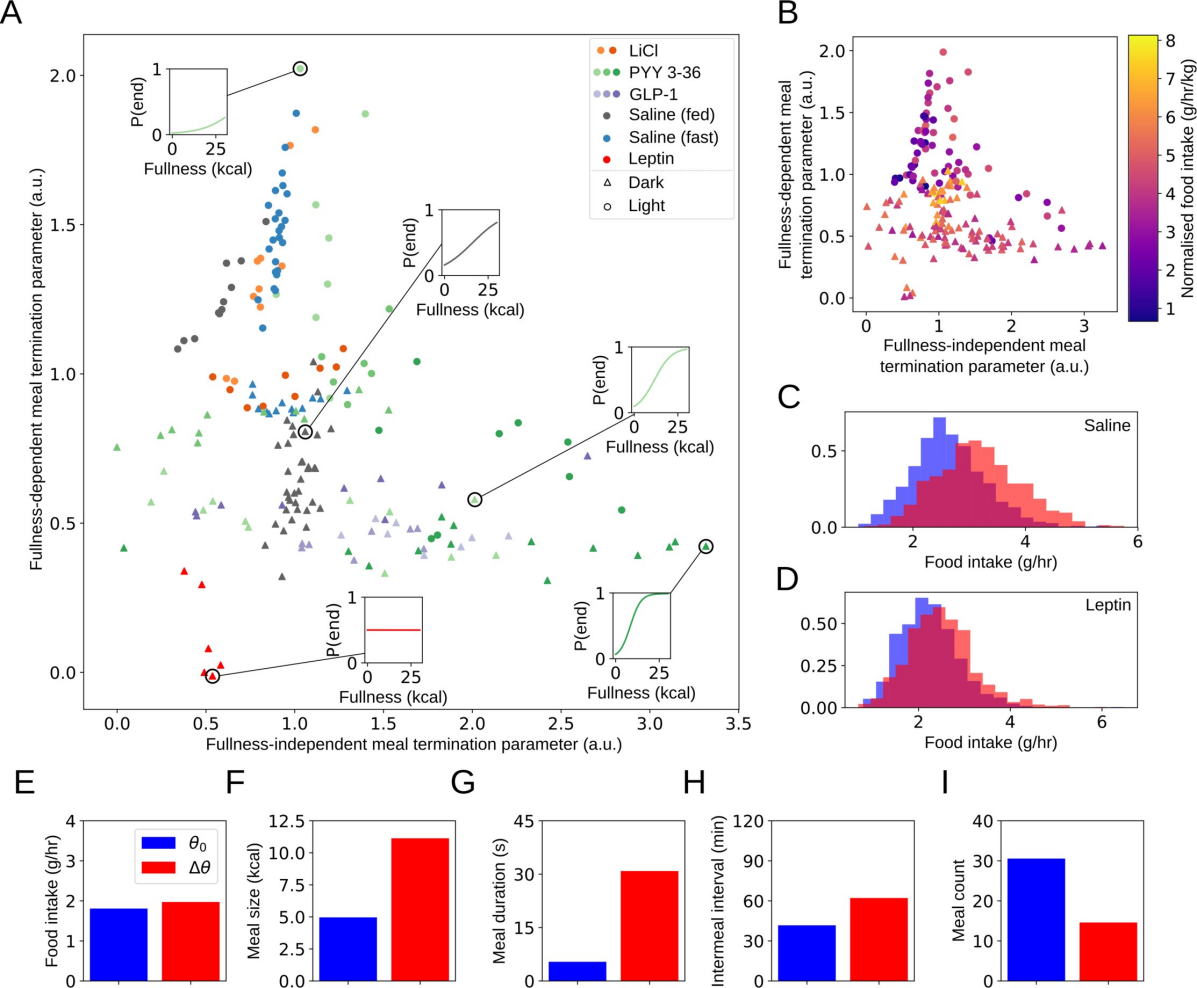
Meal termination decisions vary strongly across experimental conditions; however, the effectiveness of altering meal termination decisions to reduce food intake is complex. (A) Aversive agents (LiCl and high-dose PYY3-36) lead to meal termination at lower fullness, indicated by a move downwards and to the right. Leptin has a contrasting effect—rats given leptin are more likely to terminate meals at low fullness and are largely indifferent to fullness in their meal termination decisions. Meal termination is postponed in the dark period, consistent with increased meal size. Individual posterior mean values for fullness-independent and fullness-dependent meal termination parameters *T*_1_ and *T*_2_. Insets show posterior mean decision function for representative individuals. (B) Extreme values of either *T*_1_ or *T*_2_ are associated with decreased food intake. (C, D) Simulated food intake distributions show that the effectiveness of altering the fullness-dependent meal termination parameter *T*_2_ varies strongly with the other parameter values (baseline distribution in blue, altered parametrisation in red). Food intake is largely unchanged in simulated rats given leptin when *T*_2_ is increased; however, food intake increases substantially in simulated controls. (E–I) Alterations in multiple parameters simultaneously can produce substantial changes in the microstructure of feeding without affecting overall food intake. Altering mean feeding rate *μ*_*F*_, *T*_1_, and *T*_2_ produces an alteration of feeding microstructure similar to that seen in recent studies on CGRP neuron silencing: meals are enlarged and extended (F, G) but intermeal interval is elongated (H) and meal count decreased (I) to fully compensate over 24 hours of feeding. Underlying data for Figures A–D are available in the following repository: doi:10.17632/vpm89vrz7g.1. Inference procedure described in Materials and Methods. Details of simulation procedure for Figures E–I in S1 Text. a.u., arbitrary unit; CGRP, Calcitonin Gene-Related Peptide; GLP-1, glucagon-like peptide 1; PYY3-36, peptide YY3-36.

Next, we examined the determinants of intermeal interval duration. Our model allows for both fullness-independent and fullness-dependent control of intermeal interval duration by parameters *L*_1_ and *L*_2_, respectively, and the balance of control between these parameters varied strongly in different conditions (Fig 3A). In the dark period, mean intermeal interval was much shorter, and fullness exerted less control over intermeal interval duration. The effects of nutritional status and photoperiod were pronounced, with rats fed ad libitum in the light period having both longer intermeal intervals and much greater dependence on fullness than rats fed ad libitum in the dark period or fasted rats in either photoperiod. Our results thus show that control of meal initiation is split between longer-term energy balance (as reflected by differences in nutritional status), dynamic fullness-dependent effects, constant fullness-independent factors, and circadian rhythms. We explore possible mechanisms for fullness-dependent effects in the Discussion. Because we used a single standard chow for all experiments, the calorie content in the gut is directly proportional to weight and volume of gut contents, and so these results (and those in shown in Fig 4) would remain the same up to a rescaling of the fullness-dependent parameter. Variations in intermeal interval can have a pronounced effect on total food intake: comparing intermeal interval parameters to food intake (Fig 3B) showed that increases in food intake can be due to decreasing either the fullness-dependent or fullness-independent parameters.

PYY3-36 caused a decrease in *L*_1_ in both fasted rats in the light period and rats fed ad libitum in the dark period but showed an increase in *L*_2_ for rats fed ad libitum in the dark period only. These changes, although detectable, were small compared to the effects of nutritional status, as indicated by differences between fasted and rats fed ad libitum in both photoperiods. GLP-1, LiCl, and leptin showed minimal effects on intermeal interval at all doses, with rats fed ad libitum given either saline or one of these drugs having almost identical intermeal interval parameters (Fig 3A). This indicates that although PYY3-36 may exert anorectic effects by modulating interoceptive sensing [25], this may not be the means by which other drugs function when administered peripherally (although exogenous GLP-1 has been shown to reduce food intake primarily by reducing meal size [26]).

### Fullness at meal termination is predictive of the intermeal interval

In order to determine the validity of our conclusions on the effects of fullness on intermeal interval, we pooled data across animals in a given condition and plotted intermeal interval against our model-derived fullness variable. From this, we calculated a moving average of the intermeal interval (black line). This provides a check on our earlier conclusions that does not depend on our behavioural model. The results for rats fed ad libitum given saline in the light period are shown in Fig 3C—fullness is clearly informative of intermeal interval, and our behavioural model (red line) recapitulates the model-free estimate. Our behavioural model also permits an estimate of the degree of variability in the intermeal interval at a given value of fullness (shaded red area). The variation in intermeal interval at a given fullness is substantial, which is compatible with earlier observations that meal initiation is also controlled by a wide range of other factors [27,28].

### Fullness approaching zero is predictive of meal initiation in well-fed rats in the light period

In the light period, the mean intermeal interval closely tracked the time for fullness to reach zero, a phenomenon not observed in other conditions (see S1 Text, Section 8). This may indicate that the main signal to commence feeding in the light period is the complete emptying of the upper gut—even low values of fullness were enough to significantly reduce the probability of feeding. In the dark period, however, meal initiation probability showed a graduated response to decreases in fullness. Once again, in rats fed ad libitum, diurnal and nocturnal feeding behaviours differed dramatically, with minimal feeding in the light period and significant food intake occurring in the dark period. As with the within-bout parameters, fasting and the administration of anorectic drugs reduced the strength of this polarisation, increasing the similarity between the light and dark periods.

### Predictions of intermeal interval from fullness are more accurate than predictions from the satiety ratio

A comparison of the predicted mean intermeal interval to observed data for rats fed ad libitum in the light period showed a good agreement of model predictions with data (Fig 3C). However, both the data and the predictions showed a substantial degree of variability around the mean. This indicates that we cannot explain all of the variation in intermeal interval using fullness alone. Nevertheless, using fullness to predict intermeal interval is a substantial advance from the satiety ratio, which quantifies satiety as the ratio between the first meal size and the subsequent intermeal interval (see S1 Text, Section 7). The satiety ratio fails to give good predictions across a wide range of meal sizes (Fig 3D) for a number of reasons. Firstly, it only uses a small amount of the available data because it discards all meals other than the first. Secondly, after the first meal, fullness is poorly correlated with meal size (Fig 3E) because prior meals have often not been fully digested at meal onset. Finally, the first meal is longer than subsequent ones when recovering from a fast because it is begun with an empty upper gut, which will lead to a shorter than average intermeal interval because the rat has yet to reach its ‘typical’ fullness.

### Aversive agents and agents conventionally viewed as satiating have distinct effects on meal termination decisions

The decision to terminate a meal, also referred to as satiation, is driven by a wide range of mechanisms, including endocrine and neural signals from the stomach and small intestine [21, 22, 29], as well as gastric distention [30]. Many of these mechanisms are driven by gut-related factors, and so meal termination decisions are likely to be strongly influenced (via these pathways) by fullness. We modelled the meal termination decision as a sigmoid in fullness, with a prior that allowed for approximately linear, constant, or sigmoid variation across a physiologically plausible range of fullness. Meal termination decisions varied strongly across the data set (Fig 4A): in the light period, rats given saline had low meal termination probability and weak dependence on fullness, with the other extreme occupied by rats given high-dose PYY3-36. Both high-dose PYY3-36 and LiCl increase meal termination probability and shift termination to occurring at lower fullness, consistent with their nauseating effect [31, 32, 33]. High levels of both meal termination parameters *T*_1_ and *T*_2_ were associated with decreased food intake (Fig 4B) but for different reasons. High values of fullness-dependent termination parameters occurred for rats given saline in the light period; although these rats terminate meals later, they had much longer intermeal intervals and so experienced a net decrease in feeding compared to rats in the dark period. On the other hand, rats given PYY3-36 terminated meals at a lower fullness, consistent with a nauseating or otherwise aversive effect. This was found with high-dose PYY3-36 in both light and dark periods, but not at lower doses, suggesting that our method may be able to identify aversive agents from feeding behaviour by finding similarities in behavioural parameters. On the other hand, rats given leptin appeared almost indifferent to fullness in their meal termination decisions; they had a high probability of meal termination regardless of fullness. This is in accord with leptin reflecting long-term adipose tissue stores rather than acute nutritional status.

### Changes in meal termination can drastically change feeding microstructure without altering intake

Recent investigations into meal termination decisions have suggested that feeding behaviour is flexible enough to defend a given food intake under substantial changes to the microstructure of feeding [3,34]. Specifically, following inactivation of Calcitonin Gene-Related Peptide (CGRP)-expressing neurons in the parabrachical nucleus, both meal duration and meal size increased dramatically, with intermeal interval increasing in a compensatory fashion [3]. Similar flexibility in meal patterning in defence of a fixed food intake has also been demonstrated in mice with carnitine acetyltransferase deletions in Agouti-related peptide (AGRP)-expressing neurons [34]. We investigated whether our model was able to explain these observations by altering the meal termination parameters *T*_1_ and *T*_2_, which increase the fullness dependence of the meal termination probability and make the transition sharper, respectively (see Fig 1H). Beginning with the parameters from rats fed ad libitum given saline in the dark period, which we will denote θ_0_, we altered *T*_1_ and *T*_2_ to cause meal termination to occur at a higher typical level of fullness. In order to recover results similar to those observed in mice with inactivated CGRP neurons, it was also necessary to decrease mean feeding rate *μ*_*F*_. This is consistent with increased bout sizes shown in their representative bout data but that were not quantified. We found that under this change in parameters *θ*_0_→Δ*θ* (see S1 Text, Section 9), we were able to observe results similar to those found in mice whose CGRP neurons were ablated (see Fig 4E–4I); although the absolute values of meal size, duration, and other observables differ (as would be expected for different animals), it is possible to replicate the direction and approximate magnitude of change. This indicates the importance of understanding homeostatic regulation of food intake even on short timescales—the effect of fullness on intermeal interval duration is crucial to explaining this behavioural change and why total food intake is not reduced in these animals.

### In silico studies allow testing of novel behavioural interventions

**Fig 5:**
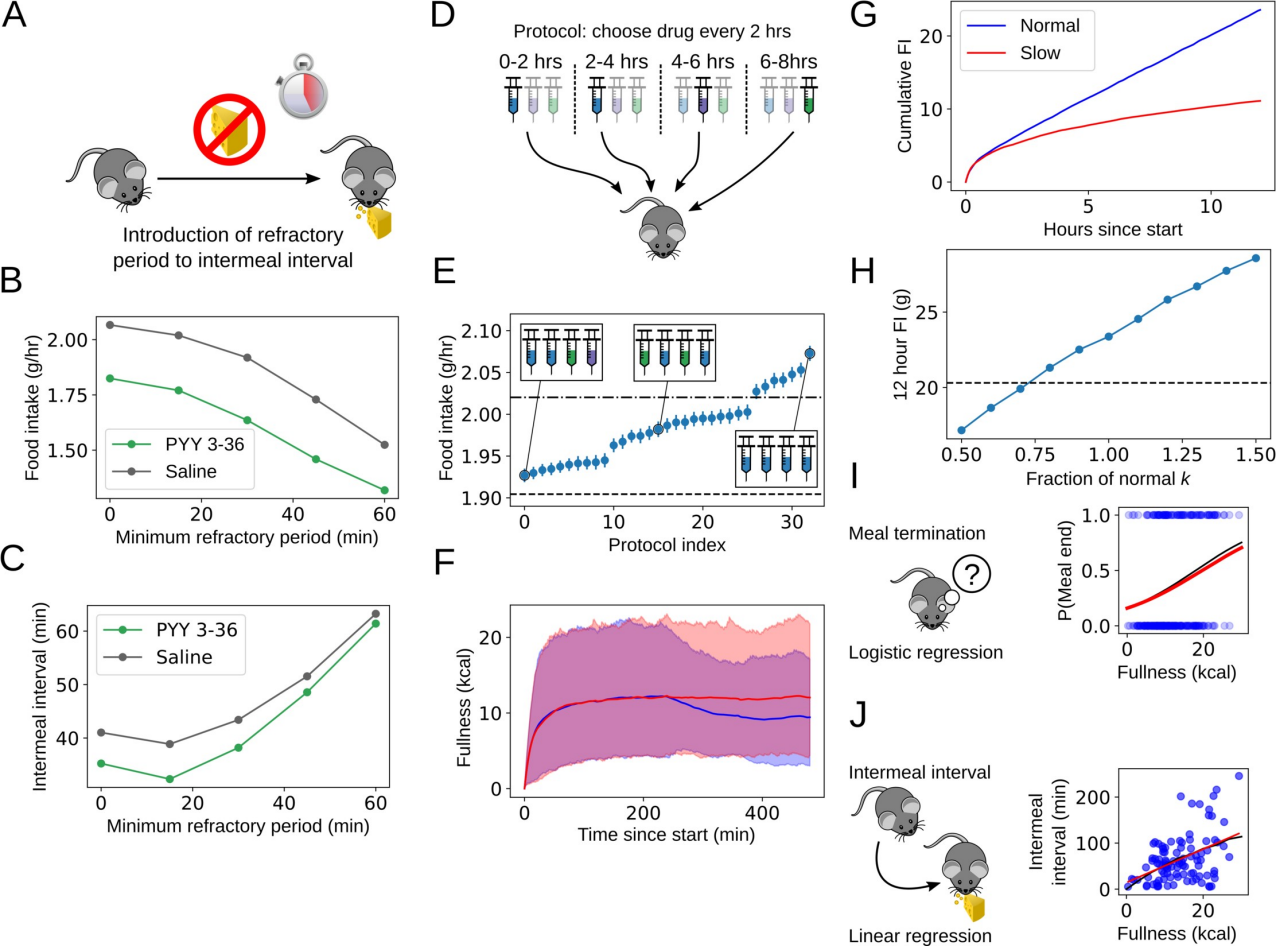
Model-based in silico experimentation allows investigation of the effects of parameter changes, design of optimal dosing protocols, and testing of behavioural interventions. (A) Schematic of refractory period experiment: following meal termination, access to food is prevented for a length of time (the refractory period). This is modelled by enforcing the intermeal interval to be at least the refractory period in length. (B) Introducing a refractory period into feeding behaviour reduces food intake to a similar degree as the administration of a high dose of PYY3-36 in simulated rats fed ad libitum in the dark period. (C) Food intake reduction occurs before the mean intermeal interval is substantially reduced, with a surprising dip in the intermeal interval when a short refractory period is introduced. (D) Schematic of optimal dosing experiment. All permutations of drug administrations, including at least 2 doses of saline, were tested with 10,000 in silico repeats. (E) Optimising drug administration schedules can reduce food intake by an additional 7% when feeding with zero initial fullness. Ad libitum-fed high-dose GLP-1, PYY3-36, and saline parameter values were used to simulate feeding behaviour with drug administration at different times. Error bars indicate standard error of the mean with 10,000 samples. Horizontal lines compare to refractory period reductions in food intake: dash-dotted and dashed lines indicate 15-minute and 30-minute refractory period food intakes for rats given saline, respectively. (F) The main effect of the optimal schedule is to reduce food intake once refed. Blue and red lines correspond to simulations using optimal administration protocol and control parameters, respectively. Shaded area indicates 95th percentile window of fullness. (G) Altering fullness decrease parameter *k* powerfully reduces food intake once refeeding is complete. A one-third reduction in *k* reduced 12-hour food intake by over 10 grams in simulated rats fed ad libitum given saline in the dark period. (H) Altering *k* over a plausible range linearly reduces food intake in simulated rats fed ad libitum given saline in the dark period. Dashed horizontal line indicates food intake typical of rats given high-dose PYY3-36. (I, J) Simplifying the model to predict meal termination with a sigmoid and intermeal interval with a linear regression (red lines) against fullness at meal termination shows good agreement with model-derived predictions (black lines). Details of simulation procedure in S1 Text. GLP-1, glucagon-like peptide 1; PYY3-36, peptide YY3-36.

In addition to exploring the effects of parameter modifications, using a generative model allowed us to test the effects of behavioural changes as well by modifying the model used to simulate behaviour. We tested the effects of introducing a ‘refractory period’ into the intermeal interval, preventing simulated rats from eating again before a length of time has elapsed (Fig 5A). In this period, meal initiation is prevented, which sets a minimum intermeal interval independent of fullness. The effectiveness of the refractory period depends on the distribution of intermeal interval durations: if the majority of intermeal intervals are longer than the refractory period, then most meal initiations will not be delayed, and food intake will not be substantially reduced. If the refractory period is longer than a substantial fraction of possible intermeal intervals, however, then the average intermeal interval will be extended, and food intake will typically be reduced. We found that introducing a 45-minute refractory period was as effective at reducing the food intake of simulated rats fed ad libitum in the dark period as high-dose PYY 3–36 (Fig 5B). We also found that the effects of combining the refractory period and PYY3-36 were additive: introducing a refractory period into the feeding of simulated rats given high-dose PYY3-36 further reduced their feeding, with the difference between rats fed ad libitum and those given PYY3-36 remaining constant as the refractory period was lengthened (Fig 5B). However, it is known that rodents can adapt over time to feeding protocols that restrict their food intake to several hours a day [35]. While it seems that multiple shorter food restricted periods throughout the day would be less likely to drive such adaptations, experimental confirmation of this would be required. Nevertheless, our model does provide an opportunity to select for further study those protocols with most potential to reduce food intake and body weight and thus to reduce the number of animal studies required.

### Optimising drug administration protocols can reduce simulated food intake

We next investigated whether a reduction in food intake could be achieved by optimising the timing of administration of anorectic drugs. Drug administration was simulated by switching to the model parametrisation corresponding to that drug for a 2-hour window. Protocols were constrained to include at least 2 doses of saline in an 8-hour period because we wanted to find the most efficient strategy in order to minimise drug administration (Fig 5D). The candidate anorectic agents were high-dose PYY3-36 and GLP-1, and parametrisations corresponding to rats fed ad libitum in the dark period were used. Unsurprisingly, the least effective strategy was to give no anorectic drugs at all. The most effective strategy consisted of administering anorectic agents at the end of the protocol, giving PYY3-36 at the 4-hour time point, followed by GLP-1 at the 6-hour time point, although variation in food intake between the 10 best protocols was modest (Fig 5E). Following the optimal administration protocol led to a reduction in mean fullness after drug administration until the end of the simulated experiment at the 8-hour time point (Fig 5F).

### A small reduction in the rate of decrease of fullness efficiently reduces food intake

Given the degree to which fullness increased intermeal interval across a wide range of conditions, it is plausible that altering gut motility (e.g., by altering food composition) could be a powerful axis for food intake reduction. Our model allows us to investigate this by altering the fullness decrease rate parameter *k* while holding behavioural parameters *θ* constant. We investigated the effects of altering *k* in rats fed ad libitum in the dark period by using the group posterior mean values of behavioural parameters. Reducing *k* to two-thirds of its normal value had a strong, long-lasting effect on 12-hour food intake but showed very little effect in the first few hours of feeding when beginning at zero fullness (Fig 5G). We then profiled the effect of changing *k* on 12-hour food intake by varying *k* between 0.5 and 1.5 times its default value. 12-hour food intake showed an approximately linear dependence on *k*, with a 20% reduction in gut emptying showing food intake reduction comparable to high-dose PYY3-36 (Fig 5H). The effects of diet composition on gut emptying has not been fully characterised; however, changes in gastric emptying of this magnitude due to changes in diet have been reported in rats [36], mice [37], and humans [38].

### A simplified version of the model can serve as an easy-to-use assay for feeding behaviour

We investigated possible simplifications of the model in order to make these tools accessible to the largest audience. The aim of this exercise was to find methods that provide most of the insight of the full model we have used in this paper, without requiring Monte Carlo model fits. We began by discarding interindividual variation by pooling data for all animals within a group before looking for simplifications of the model. A logistic regression between fullness at bout termination and meal termination probability captures the typical individual's behaviour well (Fig 5G). Because of our previous observation that the intermeal interval depends approximately linearly on fullness in most conditions (although nonlinear fits are possible in the model), we replaced the complex intermeal interval distribution (see Fig 1I) with a linear regression between intermeal interval and fullness at meal termination, which accurately reproduced the typical individual in the group (Fig 5J). Once data are pooled across individuals in a group, determining the within-bout parameters reduces to fitting exponential and normal distributions (see Fig 1). Using this information, it is possible to recover information on each of the components of feeding we have investigated in this paper in most cases, but at the expense of losing individual variation within groups. We have made code to perform these analyses available at https://github.com/tomMcGrath/feeding-behaviour.

## Discussion

We have created a model of feeding to provide a fine-grained understanding of changes in patterns of feeding behaviour and used this to reveal changes in behaviour between different photoperiods and nutritional states and under the administration of anorectic drugs. Feeding behaviour within a bout has two possible types of variation: altering bout duration and changing feeding rate. We found that there is a single axis of variation along which feeding rate increases as bout duration decreases, but the increase in feeding rate is only partially compensatory. We found that the anorectic agents GLP-1, PYY3-36, and lithium chloride mostly act on feeding behaviour within a bout and on meal termination, whereas nutritional status and photoperiod strongly affect intermeal interval as well. Leptin had no effect on bout duration or feeding rate but had a dramatic effect on meal termination consonant with its effects as a signal of adiposity. Simulation using the model allowed prediction of the effects of changes to behavioural parameters, allowing the recapitulation of recent results on CGRP neuronal ablation [3] in silico and predicting that the observed changes in meal termination and intermeal interval are accompanied by changes in within-bout feeding behaviour. Further simulations determined optimal drug administration regimes and predicted that short-term food intake restriction following meals can be as effective as strong anorectic drugs in reducing food intake, while changes to the rate at which fullness decreases may be even more effective than the anorectic agents studied here.

The neuronal circuits underlying feeding behaviour are complex. Though the roles of particular neuropeptides and classical neurotransmitters have long been recognised, more recently, the use of opto- and chemogenetic techniques has given us greater insight into the roles of specific neuronal populations. The hypothalamus and the brainstem play central roles, though other brain regions such as the thalamus and the amygdala are also considered important [39, 40]. AGRP neurons in the hypothalamic arcuate nucleus are thought to play an important in food-seeking behaviour and meal initiation, resulting in a negative-valence signal that drives animals to obtain food. However, these neurons are inactivated once food is detected, suggesting they are not responsible for consummatory behaviour [41–44]. This task is believed to fall at least partly to the lateral hypothalamus, which regulates consummatory behaviour in relation to the reward value of food [45–48]. Neurons expressing pro-opiomelanocortin (POMC) in the hypothalamic arcuate nucleus are thought to play a functionally antagonistic role to the AGRP neurons, suppressing food-seeking behaviour, food intake and body weight [49, 50], though evidence suggests that these neurons need an extended period of activation to suppress food intake and are therefore unlikely to play a major role in acute meal termination decisions [51, 52, 53]. The activity and structure of both of these arcuate neuronal populations are known to be modulated by leptin, allowing them to incorporate information about adipose tissue stores [54–57]. The circuits in the brainstem controlling meal termination and meal size are also being elucidated. Meal termination decisions have been proposed to involve signalling from the nucleus of the solitary tract to CGRP-expressing neurons in the parabrachial nucleus [3], a region that is known to incorporate quantitative information on fullness [30]. These neurons then suppress food intake via the central amygdala. Interestingly, these CGRP neurons are also regulated by projections from AGRP neurons in the arcuate nucleus, highlighting the cross talk and integration between the different brain regions involved. There is also evidence that they mediate ‘alarm’ signals and thus may respond to visceral malaise more than day-to-day ‘fullness’ [33,58]. However, inactivation of these neurons increases meal size in mice, suggesting a role in normal meal termination processes [3].

While this system is complex, it is notable that particular circuits have been implicated in three specific aspects of food intake (within-bout behaviour, meal termination, and meal initiation) consonant with both the structure of our model and our findings on the effects of anorectic agents on feeding, in that we found that these three separate feeding components were affected differently by different anorectic agents.

Although GLP-1 and PYY3-36 are both known to have acute effects on gastric emptying via the ileal brake [59] when administered peripherally, we do not model this effect. This is because their plasma half-lives are sufficiently short [60–62] that the single bolus of GLP-1 or PYY3-36 administered will be rapidly degraded, and little exogenous GLP-1 or PYY3-36 will remain for the vast majority of the experiment. If either agent were found to have a surprisingly long-lasting effect on gastric emptying, this would affect the results on satiation and satiety shown in Figs 3 and 4 but would not affect the results on ingestive behaviour shown in Fig 2. The effects of endogenous GLP-1 or PYY3-36 released postprandially are incorporated in the gut motility parameter *k*.

In this work, we have represented a complex system (the gut and its effects on behaviour) via a relatively simple model that nevertheless has predictive power. By integrating multiple factors into a single variable, we gain simplicity but lose a degree of specificity: we cannot attribute a change in behaviour to the action of a single factor (e.g., changes in postprandial cholecystokinin (CCK) release). This could be alleviated by expanding the physiological element of the model to contain multiple compartments corresponding to different anatomical components of the gut, as well as dynamical models of neural and endocrine regulatory mechanisms. Doing so would allow us to disambiguate between the effects due to stomach fullness (e.g., ghrelin release or mechanoreception) and effects due to the small or large intestine. Examples of suitable physiological models are reviewed in [63], and more continue to emerge, such as a recently developed model for ghrelin dynamics [64]. To accurately parametrise such models would require larger-scale data collection but could potentially yield new insights into the ways in which feeding behaviour is controlled.

Longer-term effects, such as gradual adaptation to sham feeding [65], could be incorporated into the framework we present here through a longer-term model of learning. The model we present here may be able to partially account for results of sham feeding experiments without further development, as in sham feeding studies, fullness will not increase during a meal, and so the probability of meal termination will remain at its lowest value throughout a meal rather than rising over the course of the meal. The probability of bout termination at zero fullness is several times lower than the probability of termination at levels of fullness that are typical at the end of meals, which would lead to meal sizes increasing substantially during sham feeding without requiring any additional mechanism. Model simulations shown in S1 Text, Section 11 indicate that sham feeding in rats fed ad libitum in the dark condition leads to an approximately 50% increase in food intake.

### GLP-1 and lithium chloride act to alter feeding behaviour within individual bouts

We found that different drugs had markedly different effects on feeding within bouts: GLP-1 and LiCl had pronounced effects on both bout duration and feeding rate, whereas the effect of PYY3-36 and leptin on these parameters was marginal at best. Interestingly, the effect of anorectic agents did not mimic the effects of ad libitum feeding in the light period. Instead of changing the values of bout duration and feeding rate towards those observed in animals fed ad libitum, they instead moved them towards the same values observed in animals given these anorectic agents in the dark period. The difference between the behaviour of animals fed ad libitum and the behaviour of those given GLP-1 and LiCl was consistent across both photoperiods: a reduction in bout duration and a counterintuitive increase in feeding rate. This suggests that the effects of the anorectic agents investigated are not to mimic the behaviour of well-fed animals but to instead create a third behavioural state that does not represent those found in natural behaviour. The fact that bout duration and feeding rate are strongly correlated suggests that they are typically regulated together because if they could be adjusted independently, then we would not expect to see the partially compensatory behaviour observed in Fig 2B.

### Fullness is predictive of intermeal interval

We found that fullness was predictive of intermeal interval in all conditions, although the degree of influence varied, and there was substantial variability in intermeal intervals at a given level of fullness. Both photoperiod and nutritional status had strong effects on satiety and changed the relationship between fullness and intermeal interval. This is consistent with previous findings, which demonstrate that meal initiation is typically under the control of learned cues [27, 28]. We speculate that we were able to observe the influence of fullness on intermeal interval because of the absence of learned cues in the environment apart from the day/night cycle (which had a pronounced effect), as well as the unchanging palatability of the chow. This means that the behaviour we observe is more akin to the ‘anticipatory/feedforward homeostatic behaviour’ discussed in [66]. This type of behaviour does not fall under the conventional definition of homeostatic behaviour because it acts to prevent a future change in a physiological variable (energy stores) rather than reacting to a perturbation that has already occurred but nevertheless allows a role for the current physiological state. A possible supplementary explanation is that previous research on the role of the gut in satiety has relied on the satiety ratio, which we show to have relatively low predictive power on our data set because it does not account for earlier meals. Our model allows for more accurate quantification of the role that the gut may play in satiety, although further research will be needed to determine the mechanisms underlying the effects we observed.

Animals with elongated intermeal intervals typically had strongly reduced food intakes. Again, nocturnal and diurnal behaviours in animals fed ad libitum represented two behavioural poles, with much longer intermeal intervals occurring in the daytime. Daytime intermeal interval duration was related to the time taken for fullness to reach zero, suggesting that in the light period, one component of feeding strategy is to wait for complete gut emptying before feeding recommences. This does not hold true in the dark period, during which intermeal intervals are much shorter. Fullness did increase intermeal interval in the dark period, indicating that although interoceptive cues were affecting feeding, the effect is less pronounced.

### The anorectic agents tested have weak or no effects on meal initiation

The anorectic drugs tested appeared to have a much less pronounced effect on the intermeal interval than they did on ingestive behaviour: PYY3-36 at moderate and high doses acted to reduce the fullness-independent component of the intermeal interval, rendering it similar in duration to intermeal intervals observed in fasted rats. On the other hand, GLP-1, LiCl, and leptin had no notable effect on these parameters (Fig 3A). It was initially surprising that leptin failed to substantially alter the intermeal interval (although it does affect meal termination—see below); however, this is likely to be because leptin was administered only to already well-fed rats, and the absence of leptin is much more effective at modulating food intake than the converse [66]. Peripheral administration of GLP-1 altered feeding within meals rather than the length of intervals between meals. This was a pharmacological study, and it is not possible to say whether GLP-1 is acting on the circuits physiologically activated by gut-derived GLP-1 or whether it is activating central circuits physiologically modulated by GLP-1–expressing neurons in the brain. Given that there are many extrinsic factors governing meal initiation [27, 67], it is perhaps unsurprising that the intermeal interval is less tightly controlled than other factors and thus less amenable to change using anorectic drugs. This may make it more easily altered by behavioural interventions, however, which we have found in silico can be as effective at reducing food intake as high doses of existing anorectic drugs. On the other hand, agents such as CCK-58 have been shown to increase the intermeal interval [68, 69] by altering the satiety ratio [69], suggesting that there is an axis for fullness-dependent regulation of the intermeal interval, even if this is typically overshadowed by other considerations. Our results support this conclusion: we found that in the majority of conditions studied, fullness was predictive of intermeal interval but that photoperiod or nutritional deficit had pronounced effects on intermeal interval.

### Leptin and high doses of LiCl and PYY3-36 have strong but opposite effects on meal termination

We observed a wide range of meal termination behaviours (Fig 4A). Rats in the light period typically were less likely to terminate meals and were less sensitive to fullness, whereas in the dark period, meal termination became much more likely and more sensitive to fullness. Unlike other behavioural parameters, nutritional status had minimal effect on meal termination, whereas anorectic agents had strong effects. High-dose lithium chloride increased meal termination probability and rendered rats more sensitive to fullness, as did high-dose PYY3-36. Low doses had much less pronounced effects, however. This is consistent with previous observations that high doses of both LiCl and PYY3-36 can be nauseating [32, 31] and so may make rats less inclined to eat large meals. Leptin's effects were distinct from both short-term nutritional status and the effects of other anorectic agents and raised the probability of meal termination while decreasing its dependence on fullness. This is in line with leptin's action as a signal of adiposity. The effects of GLP-1 on meal termination were much less pronounced, consistent with the observation that peripheral GLP-1 does not affect intermeal interval [70].

### Recent neuronal ablation studies can be replicated in silico

The structure of food intake is remarkably flexible: it can change dramatically while keeping hourly intake the same—an observation that has recently been made in the context of CGRP ablation studies [3]. We investigated whether our model could replicate this phenomenon in silico by altering the meal termination parameters *T*_1_ and *T*_2_. By altering behavioural parameters, we were able to create substantial changes in meal size and duration that left total food intake effectively unchanged (Fig 4E–4I). In order to create these changes, it was also necessary to alter mean feeding rate *μ*_*F*_, a factor that was not quantified in prior studies. This behavioural plasticity occurs because both meal termination probability and intermeal interval duration vary approximately linearly with fullness in most conditions (Figs 3 and 4). This linear variation ensures that increases in meal size are compensated for by increases in intermeal duration; increasing typical meal termination fullness by 20% leads to a 20% longer typical intermeal interval. This compensatory behaviour defends a constant food intake without needing to finely tune both intermeal interval and meal termination parameters. However, it may be possible to reduce food intake by breaking this linear relationship. This motivated an investigation of the effectiveness of introducing a brief postmeal ‘refractory period’ in which feeding is forbidden, an intervention that reduced food intake as powerfully as high doses of PYY3-36 (see below).

### Behavioural interventions targeting meal initiation may be effective

We were also able to use our generative model to perform in silico experimentation, investigating both the promise of optimising anorectic drug administration and behavioural interventions. Although it was possible to improve food intake reduction by optimising drug administration, the gains we found were relatively small. A simple behavioural intervention, on the other hand, was highly effective at reducing feeding. By introducing a ‘refractory period’ of 45 minutes during which meal initiation was forbidden, we were able to achieve food intake reduction approximately equivalent to that evoked by a high dose of PYY3-36 in the same conditions (Fig 5B).

### Decreasing gut motility potently reduces food intake

By changing the rate of decrease of fullness *k* in our simulation while keeping behavioural parameters fixed, we were able to investigate the effect of decreasing gut motility, as might be caused by altering diet composition. Physiologically plausible changes in the gut motility parameter *k* led to food intake reduction of the same or greater magnitude than that following the administration of anorectic drugs (Fig 5G and 5H). Because changes in gut motility primarily affect the intermeal interval duration, whereas we have shown that the anorectic drugs investigated here have their strongest effects on ingestive behaviour and meal termination, it is plausible that changes in gut motility could work in concert with anorectic agents to have an increased effect on food intake reduction. Both GLP-1 and PYY3-36 are believed to decrease gastric emptying rate acutely [71, 25], although our results demonstrate that this is not their only effect (and may not be their main effect long-term) because they also strongly alter feeding rate and bout duration. Our model cannot currently detect changes in gut motility rate directly from bout-level data; however, these in silico experiments indicate that reducing gut motility is a powerful way to reduce total food intake.

### Summary

We have constructed a stochastic model of feeding that accurately recapitulates observed behaviour. We validated this model and used it on a substantial data set of rat feeding in a wide variety of conditions, including across both photoperiods, both fed ad libitum and fasted, and with the administration of a variety of anorectic agents. We also demonstrated the applicability of this model to mouse feeding data with only minimal parameter changes. Using our model, we obtained deeper, more finely grained insights into the diverse behavioural effects of anorectic agents and how they reduce (or fail to reduce) food intake in different conditions. Understanding the diverse behavioural effects of anorectic agents demonstrated that they typically do not mimic ‘natural’ behaviour induced by ad libitum access to food. We speculate that it may be possible to infer characteristics of novel anorectic agents by comparing their behavioural parameters to those of well-known agents; e.g., we found that rats given LiCl and high-dose PYY3-36 occupied similar regions of parameter space, suggesting the existence of a region corresponding to aversive behaviour. We found that the precise behavioural effects of the anorectic agents we studied were as follows:

- GLP-1: reduced bout duration and increased feeding rate in rats fed ad libitum at night, no effect on meal termination or intermeal interval parameters.
- PYY3-36: diverse effects in all aspects of feeding. PYY3-36 reduced bout duration and increased feeding rate in rats fed ad libitum at night, with the opposite effect in fasted rats during the day (suggesting an alternative behavioural state not representative of typical behaviour). Meal termination occurred at lower fullness in both light and dark periods, and intermeal interval increased.
- Leptin: strong alterations in meal termination. Meal termination probability increases, especially at low fullness; no effects on other aspects of feeding.
- LiCl: decrease in feeding rate and dependence of intermeal interval on fullness. Meal termination shifted to lower fullness.

We found what appears to be a universal factor in feeding behaviour: as the duration of a bout shortens, the rate of feeding within the bout increases. This tradeoff does not perfectly maintain food intake, however, because the increase in feeding rate is insufficient to maintain the amount eaten within the bout. This observation held true across all conditions and was also observed in mice. The second reliable determinant of food intake was the factor determining how fullness affects the duration of the intermeal interval, reinforcing the necessity of using a model that tracks fullness over time. Using a dynamic calculation of fullness, rather than simply tracking the size of individual meals, led to a dramatic improvement in predictive power over the satiety ratio.

Finally, we used our computational model to perform in silico experiments investigating the effects of other interventions on food intake. We examined optimal drug administration protocols, the introduction of a postmeal refractory period, and altering gut motility. Although drug administration protocols can be optimised in silico, the effect is much weaker than either introducing a refractory period or altering gut motility. A 45-minute refractory period or a 20% decrease in gut motility were both sufficient to reduce 12-hour food intake as much as a high dose of PYY3-36, highlighting the potential of alternative interventions to reduce food intake.

We have made the code used in the analysis freely available online and have also released simplified analytical tools for easy analysis of bout feeding data. Although we believe this model is a powerful tool for gaining a finer-grained understanding of feeding behaviour, more work remains to be done to enhance the model; e.g., more detailed models might incorporate longer-term variations in behaviour due to energy balance and energy expenditure, a more complex model of the gut [72, 73], or the effects of anorectic agents on gastric emptying rate (one way by which both GLP-1 and PYY3-36 are believed to reduce food intake). The analysis we have done could also be extended to incorporate other factors we have held constant here, e.g., food palatability or environmental temperature. Further experiments investigating the effects of palatability, energy expenditure, and macronutrient composition may allow for greater insight into obesogenic behaviour. Another avenue of research is to use rich data such as videos in conjunction with bout-level feeding data to connect feeding behaviour to other aspects of behaviour, e.g., by recognising nausea from behavioural signals [74]. This could be accomplished by combining graphical models (such as the model we have used in this paper) with neural networks, an approach that has shown promise in behavioural analysis [11]. This promising avenue of research could make use of the increasing range of high-resolution data becoming available [75, 76]. Potential applications for our model could be to characterise the behavioural effects of opto- or chemogenetic changes in brain areas responsible for the regulation of food intake, to guide searches for synergistic combinations of anorectic agents, and to understand the effects of multiple treatments in combination.

## Materials and methods

### Ethics statement

Rodent studies were carried out in accordance with United Kingdom Home Office regulations and the Animals (Scientific Procedures) Act 1986 (United Kingdom) under Home Office approved project and personal licenses. Rat experiments were approved by the Imperial College Animal Welfare Ethical Review Board under Project Licenses 70/8068. Mouse experiments were approved by Home Office license (PPL: 7538 and P6C97520A) and local ethical review.

### Piecewise deterministic Markov processes

Piecewise deterministic Markov processes (PDMPs), also known as stochastic hybrid models, are a generalisation of Markov chains that capture variation in transition rates between states due to some continuously varying parameter or set of parameters [13]. The evolution of these parameters is, in turn, determined by the state of the system. In our model, the discrete states are the behavioural states (feeding *F*, short within-meal pause *S*, and long intermeal interval *L*), and the continuously varying parameter is fullness *x*(*t*). A final distinction between PDMPs and Markov chains is that the transition rate quantifies transitions out of a state, rather than into a new state.

*Fullness dynamics*. Fullness *x* is deterministic when conditioned on the behavioural state *s* ∈ {*F*, *S*, *L*} and is given by (see Fig 1)

$$\dot{x}(x,t,s)=\{\begin{array}{cc} \rho & s=F\\ -k\sqrt{x} & s=S,L \end{array},$$ (1)

where *ρ* is randomly sampled for each feeding bout (see below and Fig 1D). The parameter *k* = 0.00055 was fitted for male Wistar rats when this model was first defined [20]. Eq 1 neglects digestion during feeding to allow for a simpler mathematical formulation that yields analytically solvable transition rate equations. Feeding bouts are typically much shorter than intermeal pauses (during which the vast majority of digestion occurs), so this approximation is well-justified.

*State lifetimes*. The duration of a behavioural state *s* is a random variable whose distribution is determined by the transition rate out of that state *λ*_*s*_(*x*, *θ*), where *θ* is a vector of parameters and *x* is fullness. If the transition rate out of a state in an infinitesimal interval [*t*, *t* + *δt*] is given by *λ*_*S*_(*x*(*t*))*δt* + *o*(*δt*) and the process begins in state *x*_0_ at time *t*_0_, then the probability density function for state lifetimes is given by

$$p(t|x_{0},\theta )=\lambda_{s}(x(t,x_{0}),\theta )e^{-\int_{0}^{t}\lambda_{s}(x(\tau ,x_{0}),\theta )d\tau }.$$ (2)

Setting *λ*_*S*_ to be constant yields the exponential distribution. We define the rates as follows (see Fig 1):

$$\lambda_{F}(x)=\lambda_{F},$$

$$\lambda_{S}(x)=\lambda_{S},$$

$$\lambda_{L}(x)=\frac{1}{L_{1}+L_{2}x},$$

where the *λ*_*L*_ equation captures our intuitive understanding that feeding is more likely to recommence when fullness is zero but allows for fullness independence in the *L*_2_→0 limit, in which case the intermeal pauses will also become exponential.

*Transition kernel*. After a state finishes (e.g., the end of a bout), it is necessary to determine what happens next. The transition kernel is a parametrised function that gives the probability of transitioning from state *s* to new state *s*′ (e.g., the probability of transitioning from feeding into the within-meal pause state). The transition probabilities are

$$p(s^{\prime }|s,x,T_{1},T_{2})=\{\begin{array}{cc} 1 & s=S,s^{\prime }=F\\ 1 & s=L,s^{\prime }=F\\ \frac{1}{1+e^{-T_{1}(x-T_{2})}} & s=F,s^{\prime }=L\\ 1-\frac{1}{1+e^{-T_{1}(x-T_{2})}} & s=F,s^{\prime }=S\\ 0 & otherwise. \end{array}.$$ (3)

For clarity, deterministic transitions are not shown in Fig 1; only the stochastic transition between feeding and the pause states *S* and *F* are indicated.

### Bayesian hierarchical model

*Introduction*. In order to maximise our inferential power, as well as distinguish between interindividual and group variation, we use a Bayesian hierarchical model. This is a standard technique in Bayesian inference in which parameters for a given individual are modelled as being drawn from some distribution applying to the whole group, and we aim to infer both the individual and group parameters. Extensive explanations of this technique are available in a number of books, of which we particularly recommend [23, 24]; however, we will provide a brief introduction here.

In a hierarchical model, parameters are separated into two (or more) sets: each group (e.g., rats fed ad libitum in the dark period) has a set of hyperparameters *ψ*, which represent group-level variation, and individuals each have a set of parameters *θ*, which are drawn from *ψ*. Now, by Bayes' theorem, the posterior probability for the complete parameter set {*ψ*, *θ*} conditioned on the data *y* is given by

p(ψ,θ|y)∝p(y|θ)p(θ|ψ)p(ψ),

so the group-level variation depends on the data through the ‘lower level’ model. More concretely, in this case each individual (indexed here by *j*) has a data set we denote by *y*_*j*_. We model this data set using a PDMP with a different parameter vector *θ*^*j*^ for each individual and denote a single parameter *i* in this parameter vector by $\theta_{i}^{j}$. Group-level variation is introduced by modelling $\theta_{i}^{j}$ as being drawn from a multivariate normal distribution in a way that we will make concrete in the following subsection.

*Model formulation*. The group-level distribution was modelled as a multivariate normal with mean *μ* and covariance matrix Σ constructed by a separation strategy using an LKJ correlation matrix and independent variance parameters *τ*_*i*_:

$${\tilde{\theta }}^{j}∼N(\mu ,\Sigma )$$

Σ∼diag(τ)Ω(ν)diag(τ)

$$\tau_{i}∼\mathrm{HalfCauchy}(2.5)$$

$$\mu ∼N(\mu^{*},2)$$

where diag(*τ*) is a diagonal matrix whose elements are drawn from a half-Cauchy prior as shown above and ${\tilde{\theta }}^{j}$ is is the vector of parameters for individual *j*: ${\tilde{\theta }}^{j}=\{\lambda_{F}^{j},\rho_{F}^{j},\mu_{F}^{j},\sigma_{F}^{j},\lambda_{S}^{j},T_{1}^{j},T_{2}^{j},L_{1}^{j},L_{2}^{j}\}$. This strategy has been shown to be superior to inverse Wishart covariance matrix priors, which introduce biases into estimates of covariance [77]. The mean vector μ* = (−3,−3,−3,1,1,−1,3,3) was introduced following inspection of the ad libitum-fed saline data sets and was used in conjunction with a diffuse covariance matrix to yield a weakly informative prior that supported both strong and weak dependence of the intermeal interval duration and meal termination on fullness. With the exception of parameters *T*_1_ and *T*_2_ (the transition kernel parameters), all group-level parameters were inferred on a log10 scale.

*Inference*. We used the NUTS autotuning Hamiltonian Monte Carlo (HMC) sampler implemented in PyMC3 [78] to generate posterior samples in order to overcome the posterior curvature issues generically associated with hierarchical Bayesian models. It was also necessary to use the noncentred parametrisation suggested by Betancourt and Girolami [79] to overcome these issues. 5,000 tuning samples were generated and discarded to effectively parametrise NUTS, with 5,000 further samples taken and retained once tuning was achieved. It is worth noting that HMC samplers require a much lower sample size than traditional Metropolis–Hastings samplers because of their more rapid exploration of the posterior. MAP initialisation was not used because the typical set for hierarchical models is often far from the MAP point [79].

### Experimental procedures

*Animals*. Male Wistar rats weighing between 254 and 547 grams were individually housed under controlled temperature (21–23°C) and humidity on a 12-hour light:12-hour darkness cycle, with the light cycle between 06:00 and 18:00. All animals had ad libitum access to standard chow RM1 (SDS, Witham, UK) and water unless stated otherwise. Rats were acclimatised to all experimental procedures, including intraperitoneal injection. All animal procedures were in accordance with the UK Home Office Animals (Scientific Procedures) Act 1986 and were approved by the Animal Welfare and Ethics Review Board at the Central Biological Services unit at the Hammersmith Campus of Imperial College London.

*Feeding data collection and preprocessing*. Rats were individually placed into a 24-chamber open-circuit CLAMS (Columbus Instruments, Columbus, OH, USA) and acclimatised to the system for 24 hours prior to fasting. In protocols 1 and 2 (shorter-duration studies involving the administration of anorectic drugs), animals were acclimatised to intraperitoneal injections prior to the experiment. Quantitation of feeding was carried out using the feeding bout data reported by CLAMS. This was processed prior to analysis in order to remove erroneous data (negative and cancelling readings, low and high outliers), as described in S1 Text, Section 2. This processing can be reproduced using the data and notebooks available at https://github.com/tomMcGrath/feeding-behaviour. The number of animals reported in Table 2 is the number remaining after data cleaning (see S1 Text, Section 2).

*Protocol 1*. Animals were allowed to feed ad libitum both prior to and throughout the experiment. In the early dark period, anorectic drugs were administered via intraperitoneal injections at the doses and times in Table 2.

*Protocol 2*. Following an overnight fast, animals received an intraperitoneal injection of saline or an anorectic drug (see Table 2) and were subsequently allowed to feed ad libitum from the early light period. Injections were carried out according to the schedule below, allowing for the study of feeding behaviour in both photoperiods.

*Protocol 3*. Following an overnight fast, animals were allowed to feed ad libitum from the onset of the light period. Data from this protocol were recorded as a continuous 72-hour recording and segmented according to the times in Table 2. After 24 hours of ad libitum feeding following the overnight fast, rats were assumed to be refed and so were classified as ad libitum-fed for the remaining two light/dark cycles.

*Drug administration*. Anorectic drugs, doses, and administration schedules are given in Table 2.

## Supporting information

S1 Text Supplementary details for Supplementary Figures. (PDF)

S1 Fig Anorectic agents reduce food intake in a dose-dependent manner. Dark dashed line separates light and dark period data, and light dashed lines separate different anorectic agents. Error bars show standard error of the mean. ‘R’ in group label denotes refeeding from a fast, ‘A’ denotes rats fed ad libitum. ‘L’ denotes light period feeding, and ‘D’ denotes dark period.
(TIF)

S2 Fig The stochastic feeding model can also be applied to mouse data. Right column indicates bodyweight-normalised food intake. (A, B) The imperfect duration/feeding rate tradeoff appears in both the dark and light period for mice. (C, D) Mice show a wide degree of variation in *L*_2_. (E, F) Meal termination parameters are correlated in mice, and low *T*_1_ and *T*_2_ are both associated with high food intake.
(TIF)

S3 Fig Group-level plots for characteristic bout duration and feeding rate. Symbol type indicates photoperiod. Triangles: dark period, circles: light period. (A) Fasted and ad libitum-fed rats given saline in the dark and light periods. (B) Rats given PYY3-36 in the light (fasted) and dark (ad libitum-fed) periods. (C) Lithium Chloride recovering from a fast in the light period. (D) Rats fed ad libitum given GLP-1 in the dark period. (E) Rats fed ad libitum given leptin in the dark period. GLP-1, glucagon-like peptide 1; PYY3-36, peptide YY3-36.
(TIF)

S4 Fig Intermeal interval tracks fullness reaching zero in rats fed ad libitum in the light period but not the dark. Intermeal interval tracks fullness reaching zero in rats fed ad libitum in the light period (A) but not the dark (B). Blue circles indicate intermeal interval data, red line indicates the mean intermeal interval (obtained from Monte Carlo simulation), and the dashed line shows the time for fullness to reach zero if no feeding took place.
(TIF)

S5 Fig Our model is robust to changes in fullness dynamics. (A) Intermeal interval parameters inferred using a modified model of fullness are not substantially changed from the model used in the main text (c.f. Fig 3A). (B) Meal termination parameters are minimally changed under the modified model of fullness (c.f. Fig 4A).
(TIF)

S6 Fig Simulated sham feeding increases food intake by approximately 50%. Error bars indicate standard error of the mean over 10,000 samples.
(TIF)

## Abbreviations

AGRP: Agouti-related Peptide
a.u: arbitrary unit
CCK: cholecystokinin
CGRP: Calcitonin Gene-Related Peptide
CLAMS: Comprehensive Lab Animal Monitoring System
GLP-1: glucagon-like peptide 1
HMC: Hamiltonian Monte Carlo
IMI: inter-meal interval
POMC: pro-opiomelanocortin
PYY3-36: peptide YY3-36
