# Supplementary material for: The homeostatic dynamics of feeding behaviour identify novel mechanisms of anorectic agents
Source: PLoS Biol. 2019 Dec 5;17(12):e3000482. doi: 10.1371/journal.pbio.3000482 (PMC6894749; doi:10.1371/journal.pbio.3000482)
Supplement: S1 Text — (PDF) [file pbio.3000482.s001.pdf]

The homeostatic dynamics of feeding behaviour identify novel mechanisms of anorectic agents - Supplementary Material

Thomas M McGrath, Eleanor Spreckley, Aina Fernandez Rodriguez, Carlo Viscomi, Amin Alamshah, Elina Akalestou, Kevin G Murphy, Nick S Jones

1 Data and software availability

Raw and preproccessed data is available as a Mendeley dataset (`doi:10.17632/vpm89vrz7g.1`), and all of the code used to process the data, perform inference and generate figures is available as a Github repository at `https://github.com/tomMcGrath/feeding-behaviour`.

2 Data preprocessing

The CLAMS system used measures feeding data from a centre feeder. Bout size is determined by changes in feeder weight, which is susceptible to two main sources of error: spillage and animals standing on the feeder. In order to account for these sources of error, bout data was cleaned according to the following criteria:

- Bout size $<$ 4 grams
- Bout duration $<$ 1000 seconds
- Feeding rate $<$ 0.02 grams per second
- Bout duration $>$ 4 seconds.

Bouts not meeting these criteria were removed from the dataset. In addition, bouts of negative size and bouts that were immediately cancelled by a subsequent bout were also removed - the latter were assumed to arise from the animal standing on the scales. Data for a rat was taken to be unreliable and removed if any of the following criteria held:

- More than 25 cancelling bouts
- More than 200 negative-value bouts

- Less than 5 bouts
- More than 30 bouts excluded by the bout cleaning criteria above.

This procedure led to removal of 43 out of 198 files from the original dataset.

3 Model validation via simulation

The groups used in model design were low, medium and high-dose PYY given to *ad libitum* fed rats in the dark period, and their associated controls. All other data was held out and not used in the model design phase. Priors were obtained via Empirical Bayes from this dataset. Simulations were performed by Monte-Carlo resampling the individual posterior parameter distribution for each rat 100 times, matching initial fullness and behaviour sequence duration. The mean of the simulated food intake distribution was compared against the actual food intake in order to produce Fig 1H.

4 Reduction of food intake by anorectic agents

S1 Fig shows average normalised food intake for each group. In order to account for differences in bodyweight and experiment duration between experimental groups, food intake was normalised to units of grams per hour per kilogram of bodyweight. Bodyweight values were recorded at the beginning of each experiment. Rats recorded as recovering from a fast in the dark period were subject to an overnight fast before refeeding was permitted in the light period. Although feeding was elevated following the overnight fast (S1 Fig), normalised FI was lower than dark period values, so we still consider these rats to be in a nutritional deficit.

5 Inference for mouse data

5.1 Experimental procedures

Mouse behavioural data was obtained from both male and female wild-type C57BL/6 mice between 17.8 and 36.2 grams in the light and dark periods. Light hours were 0600-1800. Animals were allowed *ad libitum* access to standard chow (R105, Safe Diets, Augy, France) throughout. Mice were acclimatised to all experimental procedures. All procedures were conducted under the UK Animals (Scientific Procedures) Act, 1986, approved by Home Office license (PPL: P6C97520A) and local ethical review. The animals were maintained on a C57BL/6 background at 19-21 C in a temperature- and humidity-controlled animal-care facility, with a 12 hr light/dark cycle and free access to water and food.

5.2 Data preprocessing

Data preprocessing was carried out using the same code as the rat data, with the following changes in exclusion criteria due to the increased length of the behaviour sequences:

- More than 50 cancelling bouts
- More than 200 negative-value bouts
- Less than 5 bouts
- More than 200 bouts excluded by the bout cleaning criteria above.

5.3 Data analysis

Group level priors were set via Empirical Bayes analysis of fully pooled data. Based on this analysis, group means were set to:

$$\theta = [-2, -4, -4, 1, 1, -1, 3, 4] \tag{1}$$

To accomodate the decreased size of mouse guts, we reparametrised the meal termination function to

$$\text{Prob}(end) = (1 + \exp(-0.1T_1(x - T_2))^{-1}. \tag{2}$$

Apart from these change in parameters, inference was carried out using the same code as the rat data (see Materials and Methods), using the NUTS sampler with 5,000 tuning and 5,000 sample steps.

5.4 Results

See S2 Fig A-F.

6 The bout duration/feeding rate tradeoff holds within groups

In order to ensure that the characteristic bout duration/mean feeding rate tradeoff we observed held both within and across groups, we examined feeding data for each anorectic agent separately. With the exception of leptin, which shows limited variation in either parameter, this trend held consistently across different conditions (S3 Fig).

7 Calculating the satiety ratio

The satiety ratio was calculated for each group using the first meal from each animal's data. Meals were determined using a hard cutoff in order to avoid including assumptions from our Bayesian model: bouts followed by pauses of less than 300 seconds were grouped into meals. The group satiety ratio was calculated as the average of the individual satiety ratios - for a group of size N, the satiety ratio r is given as

the average of the individual satiety ratios r_i. These are determined from the first meal size s_i^0 and intermeal interval t_i^0

$$r = \frac{1}{N}\sum_{i=1}^{N} r_i = \frac{1}{N}\sum_{i=1}^{N} \frac{s_i^0}{t_i^0}.$$

Intermeal interval predictions $\tilde{t}_i$ were generated for subsequent meals of size s_i using the formula $\tilde{t}_i = r s_i$.

8 Mean intermeal interval tracks time for fullness to reach zero in the light period

Emptying time t_0 was calculated using the formula $t_0 = 2\sqrt{x_0}/k$, where x_0 is fullness at the beginning of the intermeal interval and the parameter k determines the rate at which fullness decreases. These were compared to intermeal intervals sampled using the group mean parameters for *ad libitum* fed rats in the light and dark period to generate S4 Fig. The light period shows nonlinear dependence of intermeal interval on initial fullness in a way that matches the time for fullness to reach zero, whereas the dark period shows approximately linear dependence on initial fullness.

9 Replicating CGRP ablation studies *in silico*

We obtained the perturbed parameter set $\Delta\theta = \theta_0 + \theta_p$ from the group-level posterior mean values θ_0 of *ad libitum* fed rats given saline in the dark period. We used a perturbation $\theta_p = [0, -0.5, 0, 2, 1, 0, 0, 0]$ to perturb only the meal termination and feeding rate parameters. We took 100 sample trajectories from the model and averaged them in order to obtain Figs 4 E-I.

10 Results are robust to a modification of the fullness model

In order to determine the extent to which our results are dependent on the nature of our fullness model we performed the same analysis except with a modified fullness model. In this model, the rate of decrease is linear in current fullness x

$$\dot{x}(x,t,s) = \begin{cases} \rho & \text{s = F} \\ -\tilde{k}x & \text{s = S, L.} \end{cases} \tag{3}$$

Under this model, fullness will decrease exponentially ($x(t) = x_0 e^{-\tilde{k}t}$) and so will never reach zero. We chose the parameter $\tilde{k}$ such that the time for fullness to reach half of its initial value matched Equation 1 for a reference initial fullness $\tilde{x}_0$. Enforcing this criterion leads to the condition

$$\tilde{k} = \frac{k \ln(2)}{2\sqrt{\tilde{x}_0}(1 - 2^{-1/2})}. \tag{4}$$

We set $\tilde{x}_0 = 10$ (a typical fullness value), leading to $\tilde{k} \approx 0.37k$. Substituting Equation 3 into the likelihood for the intermeal interval yields a likelihood for the modified model. We performed the same inference procedure as for the model used in the rest of the paper, except with the standard deviation for the prior over group mean set to 1 in order to alleviate numerical instabilities during sampling.

The results are shown below (S5 Fig). We show the effects on both the satiety and satiation parameters, as these both have dependence on the fullness parameter. Ingestive behaviour (feeding rate and bout duration) is not fullness-dependent in our model, and so is not affected by the change of model. S5 Fig A shows good qualitative agreement with Fig 3A, although low values of L_1 have increased in S5 Fig A. A decrease in L_1 from $L_1 \approx 1$ to $L_1 \approx 0$ leads to only minimal changes in behaviour at the values of L_2 we find, as the L_2 term in the likelihood is much larger than the L_1 term for typical values of x. Thus, although some parametric differences exist, these are not behaviourally significant. S5 Fig B shows strong agreement with Fig 4A, further demonstrating the robustness of our model.

11 Simulated sham feeding results in an approximately 50% increase in meal size

We simulated sham feeding by sampling meals from the posterior predictive distribution without increasing the fullness variable during feeding. We drew 10,000 samples from the posterior distribution *ad libitum* fed rats in the dark period, sampled a meal from each draw, and recorded the meal size. Normal meals were sampled identically, except that fullness was updated throughout the meal. Meals were started with zero initial fullness to mimic recovery from a fast. S6 Fig shows an approximately 50% increase in feeding in the sham condition.
