# Supplementary figures and images for: The homeostatic dynamics of feeding behaviour identify novel mechanisms of anorectic agents
Source: PLoS Biol. 2019 Dec 5;17(12):e3000482. doi: 10.1371/journal.pbio.3000482 (PMC6894749; doi:10.1371/journal.pbio.3000482)

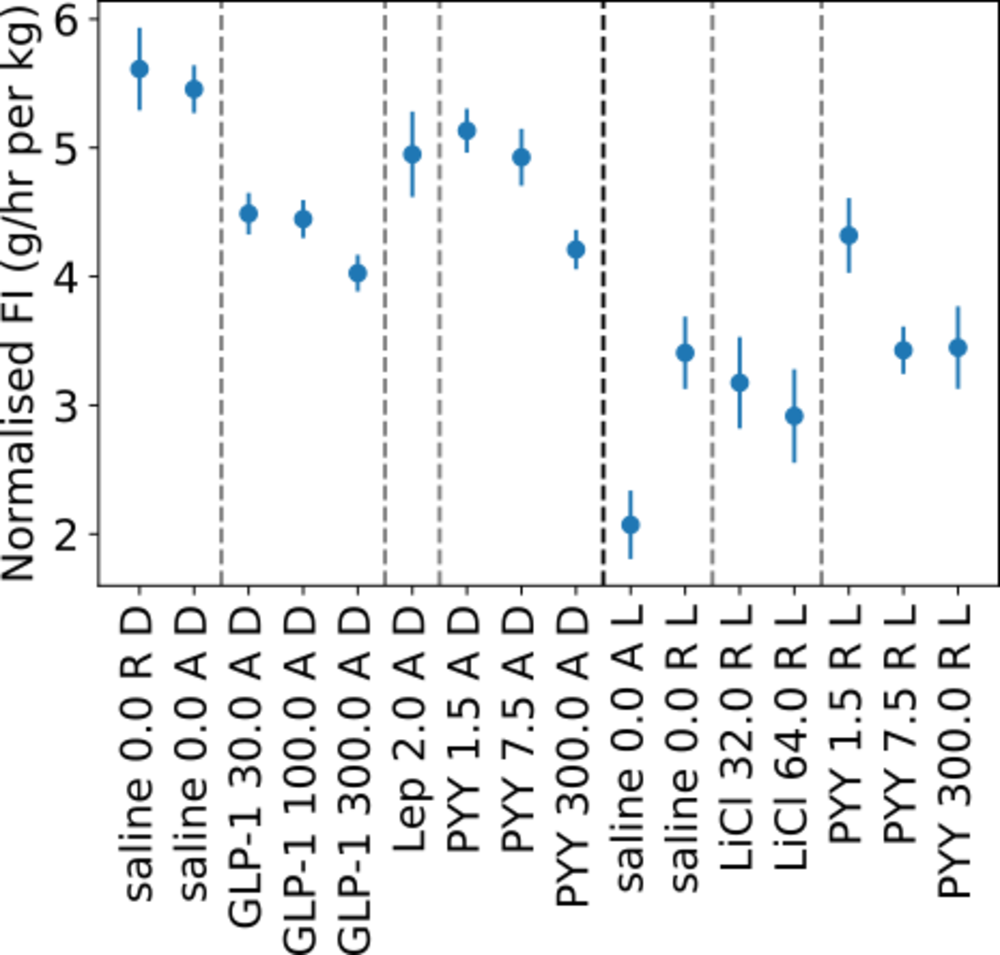

Supplement: S1 Fig — Dark dashed line separates light and dark period data, and light dashed lines separate different anorectic agents. Error bars show standard error of the mean. ‘R’ in group label denotes refeeding from a fast, ‘A’ denotes rats fed ad libitum. ‘L’ denotes light period feeding, and ‘D’ denotes dark period. (TIF) [file pbio.3000482.s002.tif]

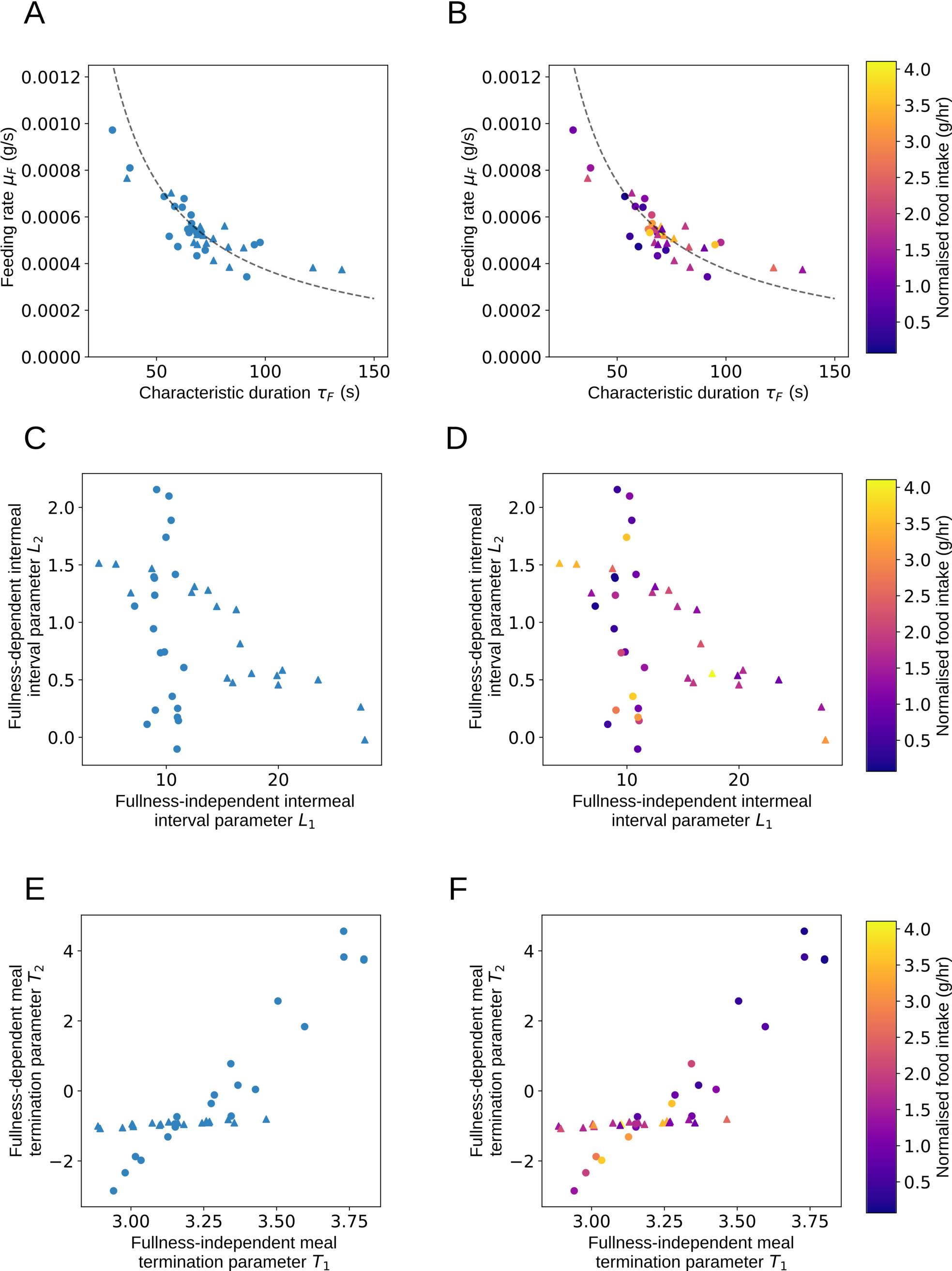

Supplement: S2 Fig — Right column indicates bodyweight-normalised food intake. (A, B) The imperfect duration/feeding rate tradeoff appears in both the dark and light period for mice. (C, D) Mice show a wide degree of variation in L2. (E, F) Meal termination parameters are correlated in mice, and low T1 and T2 are both associated with high food intake. (TIF) [file pbio.3000482.s003.tif]

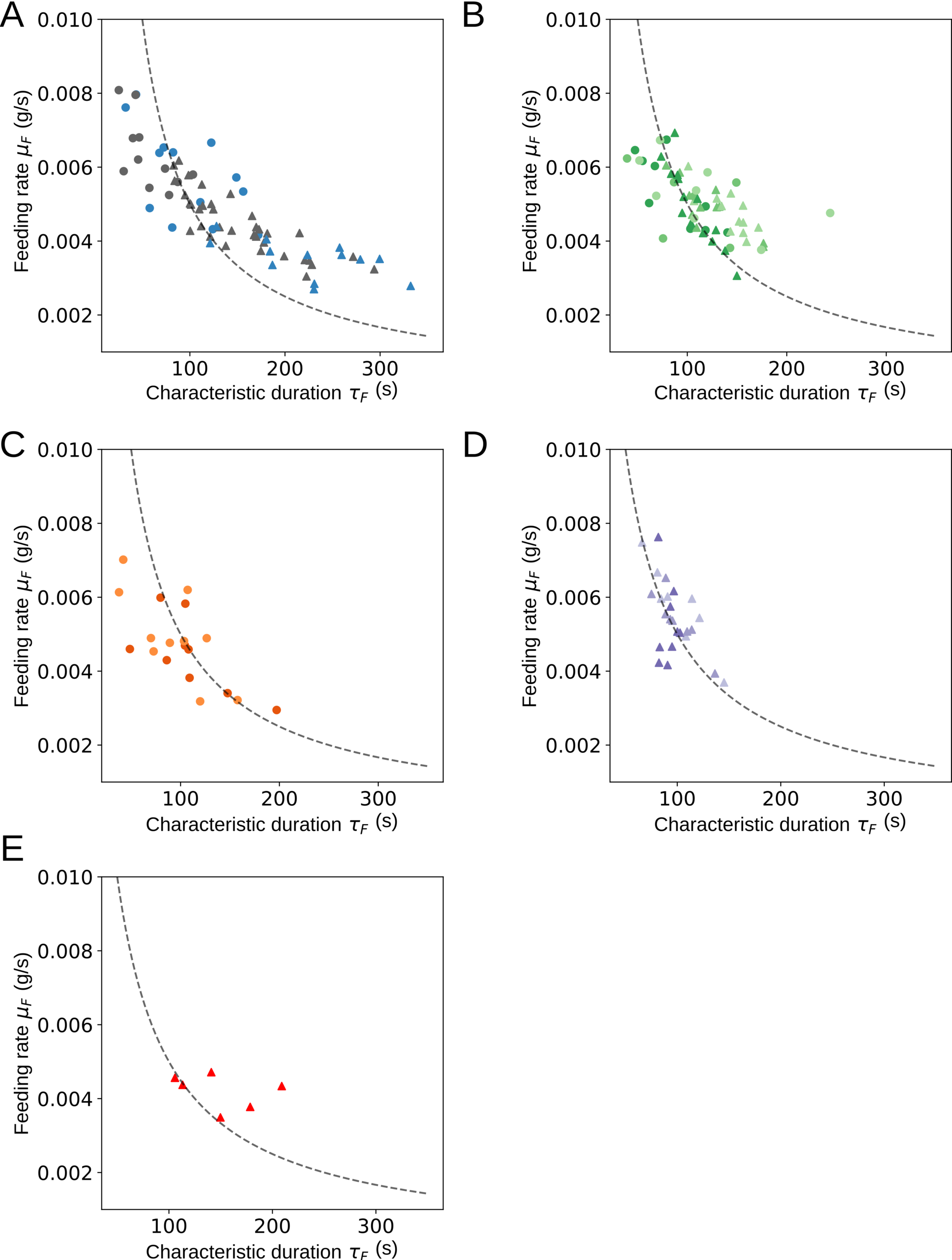

Supplement: S3 Fig — Symbol type indicates photoperiod. Triangles: dark period, circles: light period. (A) Fasted and ad libitum-fed rats given saline in the dark and light periods. (B) Rats given PYY3-36 in the light (fasted) and dark (ad libitum-fed) periods. (C) Lithium Chloride recovering from a fast in the light period. (D) Rats fed ad libitum given GLP-1 in the dark period. (E) Rats fed ad libitum given leptin in the dark period. GLP-1, glucagon-like peptide 1; PYY3-36, peptide YY3-36. (TIF) [file pbio.3000482.s004.tif]

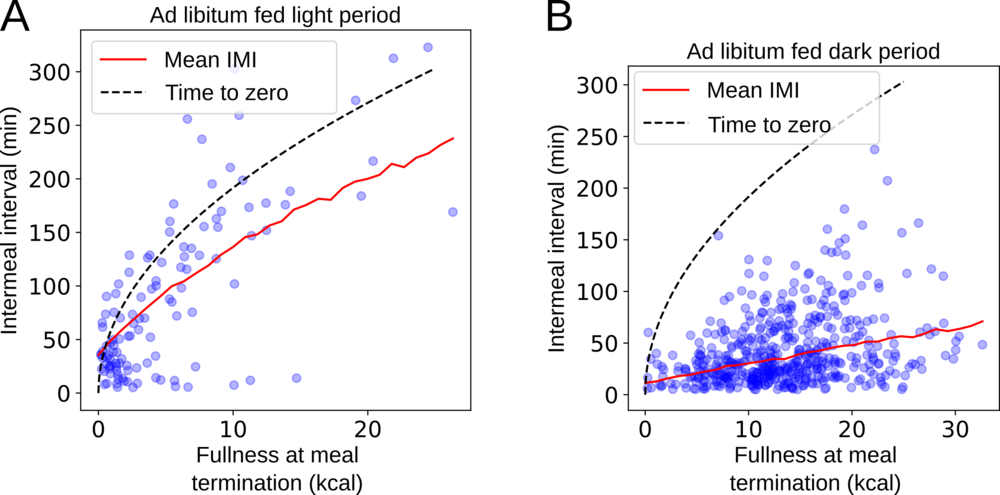

Supplement: S4 Fig — Intermeal interval tracks fullness reaching zero in rats fed ad libitum in the light period (A) but not the dark (B). Blue circles indicate intermeal interval data, red line indicates the mean intermeal interval (obtained from Monte Carlo simulation), and the dashed line shows the time for fullness to reach zero if no feeding took place. (TIF) [file pbio.3000482.s005.tif]

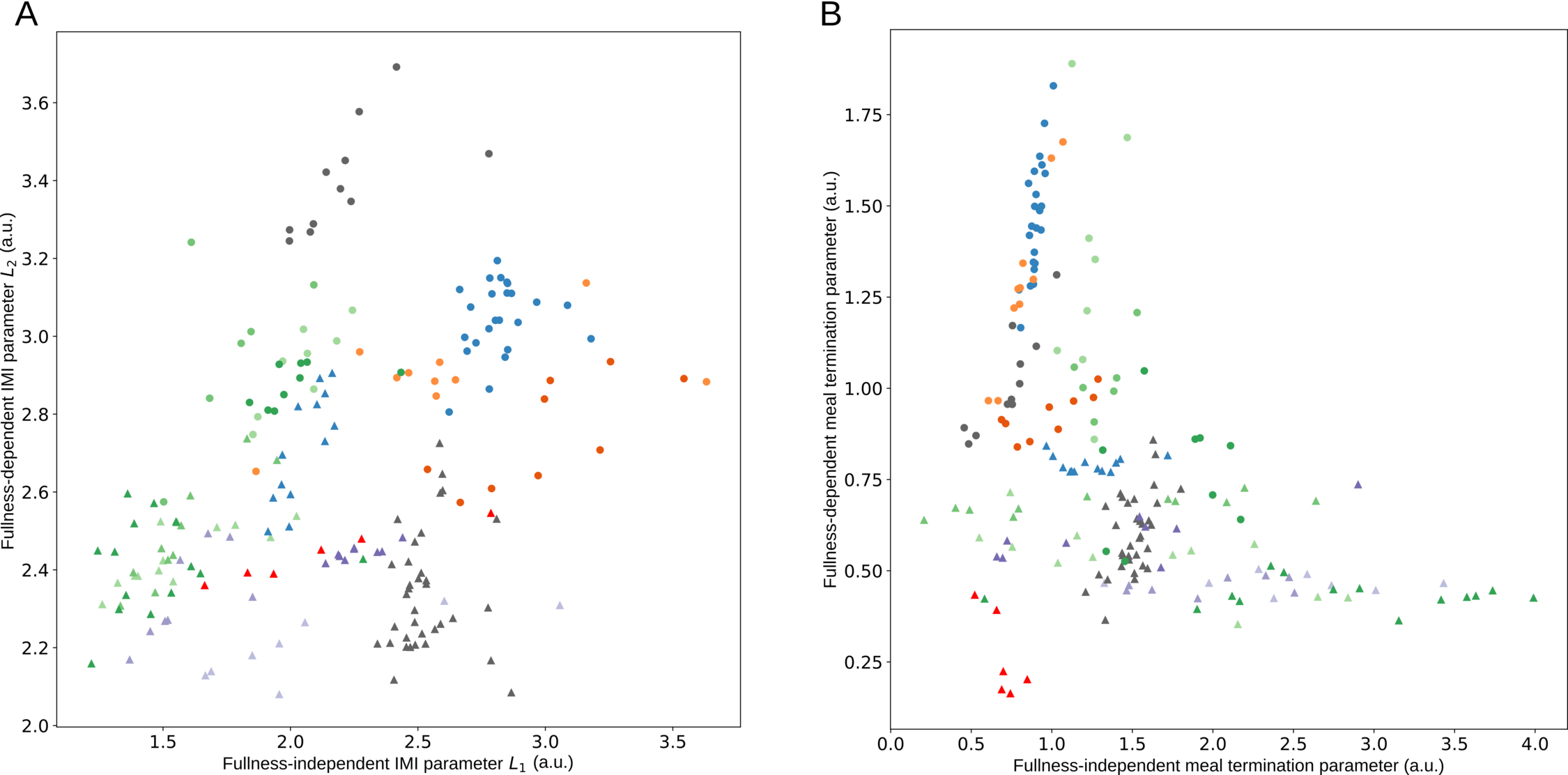

Supplement: S5 Fig — (A) Intermeal interval parameters inferred using a modified model of fullness are not substantially changed from the model used in the main text (c.f. Fig 3A). (B) Meal termination parameters are minimally changed under the modified model of fullness (c.f. Fig 4A). (TIF) [file pbio.3000482.s006.tif]

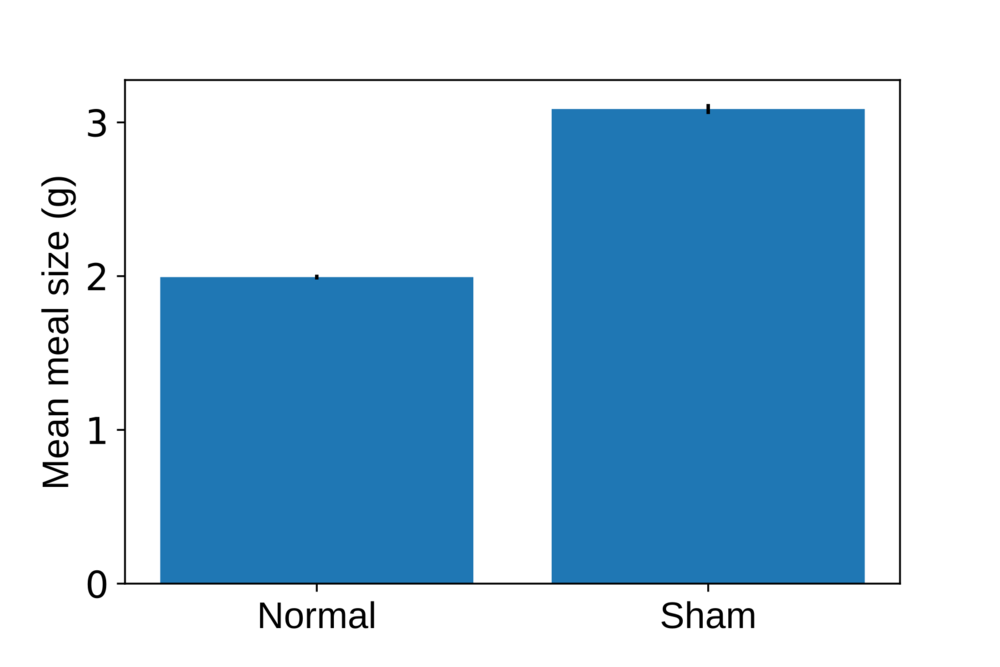

Supplement: S6 Fig — Error bars indicate standard error of the mean over 10,000 samples. (TIF) [file pbio.3000482.s007.tif]
